# Rational design of an “all-in-one” phototheranostic†

**DOI:** 10.1039/d0sc03368e

**Published:** 2020-07-21

**Authors:** Zi-Shu Yang, Yuhang Yao, Adam C. Sedgwick, Cuicui Li, Ye Xia, Yan Wang, Lei Kang, Hongmei Su, Bing-Wu Wang, Song Gao, Jonathan L. Sessler, Jun-Long Zhang

**Affiliations:** Beijing National Laboratory for Molecular Sciences, State Key Laboratory of Rare Earth Materials Chemistry and Applications, College of Chemistry and Molecular Engineering, Peking University Beijing 100871 P. R. China zhangjunlong@pku.edu.cn; Department of Chemistry, The University of Texas at Austin 105 East 24th Street-A5300 Austin TX 78712-1224 USA sessler@cm.utexas.edu; Department of Nuclear Medicine, Peking University First Hospital Beijing 100034 P. R. China kanglei@bjmu.edu.cn; College of Chemistry, Beijing Normal University , Beijing 100875 P. R. China; School of Chemistry and Chemical Engineering, South China University of Technology Guangzhou 510640 P. R. China

## Abstract

We report here porphodilactol derivatives and their corresponding metal complexes. These systems show promise as “all-in-one” phototheranostics and are predicated on a design strategy that involves controlling the relationship between intersystem crossing (ISC) and photothermal conversion efficiency following photoexcitation. The requisite balance was achieved by tuning the aromaticity of these porphyrinoid derivatives and forming complexes with one of two lanthanide cations, namely Gd^3+^ and Lu^3+^. The net result led to a metalloporphodilactol system, Gd-*trans*-**2**, with seemingly optimal ISC efficiency, photothermal conversion efficiency and fluorescence properties, as well as good chemical stability. Encapsulation of Gd-*trans*-**2** within mesoporous silica nanoparticles (MSN) allowed its evaluation for tumour diagnosis and therapy. It was found to be effective as an “all-in-one” phototheranostic that allowed for NIR fluorescence/photoacoustic dual-modal imaging while providing an excellent combined PTT/PDT therapeutic efficacy *in vitro* and *in vivo* in 4T1-tumour-bearing mice.

## Abstract 2

We report here porphodilactol derivatives and their corresponding metal complexes as “all-in-one” phototheranostics by controlling the relationship between intersystem crossing (ISC) and photothermal conversion efficiency following photoexcitation.

## Graphical abstract

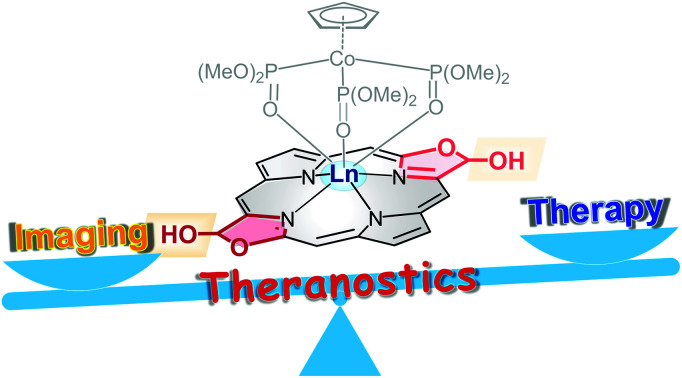

## Introduction

Theranostics (or theragnostics) are emerging as an attractive alternative to the classic “one medicine fits all” approach to disease management.^1,2^ The combination of both diagnostic and therapeutic components into one single system provides a strategy that can image diseased tissue, monitor drug delivery, and evaluate therapeutic efficacy. This provides the ability to tailor treatments to an individual patient (personalised medicine).^3^ Owing to the non-invasive, high precision and controllable nature of light, optical-based phototheranostics have garnered increasing attention of late.^4–6^ In these systems, the near-infrared (NIR) optical window (650–1700 nm) has been frequently targeted in an effort to achieve desirable deep tissue light penetration and high resolution imaging with good sensitivity.^7,8^ Previous systems have adopted an “assembly” approach that combines various imaging/therapeutic components into one platform.^9–11^ Although an attractive strategy, the inherent complexity and the potential for unknown toxicities could serve as impediments to clinical translation.^12–14^ Single molecule-based phototheranostics with various imaging and therapeutic properties could prove easier to prepare and use. However, such systems are under-explored.^15–20^ In this study we use a combination of structural tuning and control over excited state energetics to create a porphyrinoid derivative, Gd-*trans*-**2**, that shows promise as an “all-in-one” phototheranostic. As detailed below, Gd-*trans*-**2** permits NIR fluorescence/PA imaging for tumour diagnostics and provides for PTT/PDT efficacy *in vitro* and *in vivo*.

**Scheme 1:**
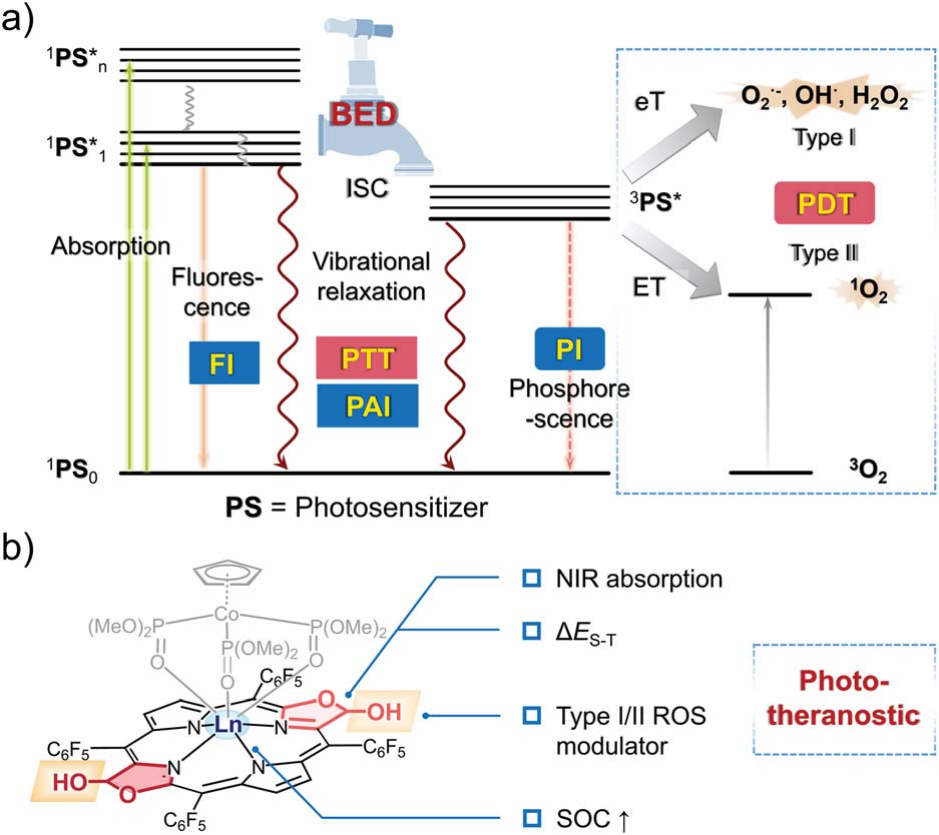
The balanced energy dissipation (BED) approach to creating a phototheranostic agent explored in this study. (a) Jablonski diagram showing the various energy dissipation pathways of the excited states and the corresponding phototheranostic functions, including optical imaging (fluorescence imaging (FI) and phosphorescence imaging (PI)), photoacoustic imaging (PAI), photothermal therapy (PTT), and photodynamic therapy (PDT, Type I and Type II). (b) Generalized structure of a bacteriochlorin-based porphodilactol phototheranostic agent.

Controlling the excited state features was considered key to the successful design of Gd-*trans*-**2**. In general, when a fluorophore is promoted to its excited state, there are a number of ways for the energy to dissipate. This includes the emission of light, intersystem crossing (ISC) and non-radiative relaxation. In terms of phototheranostics, these dissipation pathways can result in favourable fluorescence (FI), phosphorescence (PI) and photoacoustic imaging (PAI) properties, as well as the ability to promote desirable light-based outcomes, such as photodynamic therapy (PDT) and photothermal therapy (PTT) (Scheme 1a).^21^ Each pathway is connected to one another and can be modulated through the population of an appropriate excited state. Therefore, the ability to control the dissipation of excited state energy within a given putative phototheranostic might allow it to be tailored to a specific application and ultimately the benefit of individual patients. However, to the best of our knowledge, there are few clear design criteria that allow for productive correlations between molecular structure and the corresponding dissipation of excited state energy. A goal of the present study was to achieve such control as embodied in the preparation of a potentially useful phototheranostic.

A predicative requirement for an effective phototheranostic is high molar absorptivity in the NIR region. Next, an appropriate population of the singlet and triplet excited states is required. The associated balance is typically determined by the extent of ISC, a key process that obeys Fermi's golden rule.^21,22^ The efficiency of ISC is dependent upon the spin–orbit coupling (SOC) matrix element (〈T_i_|H_SO_|S_1_〉) and the energy gap between the lowest singlet excited state and the corresponding triplet excited state (Δ*E*_S–T_).^23^ To be effective, phototheranostics generally incorporate useful photodynamic properties. In this context, it is important to consider so-called Type I or Type II pathways for the production of reactive oxygen species (ROS). Type II relies on the production of singlet oxygen. In contrast, Type I activation produces radicals directly and could thus allow for the creation of effective PDT systems that operate in hypoxic environments (*e.g.*, the interior of tumours).^24,25^

Bacteriochlorin represent a class of easy to derivatise and naturally occurring tetrapyrrole cofactors with excellent NIR absorptivity^26,27^ that have shown potential as PDT agents.^28^ However, few have been reported as theranostic agents.^29^ Over the years, we have been interested in modulating the aromaticity of these tetrapyrrole cofactors and evaluating their photophysical properties.^30–33^ In this work, we show that it is possible to balance the energy dissipation (BED) between excited states in appropriately functionalised lanthanide(iii) bacteriochlorin derivatives as a rational approach to creating “all-in-one” phototheranostics (Scheme 1b). This effort culminated in the preparation of the gadolinium(iii) porphodilactol Gd-*trans*-**2**, a species that could be incorporated into mesoporous silica nanoparticles (MSN) to generate constructs that permits the fluorescence/PA monitoring of PTT/PDT-based treatments of 4T1-tumour-bearing mice.

## Results and discussion

### Design and synthesis of photosensitisers

Porpholactones represent a class of synthetic porphyrinoids, in which a β-pyrrolic double bond is replaced by a lactone moiety.^34^ The resulting oxazolone unit affords a porphyrinoid-based platform from which a library of tetrapyrrole-based biomimetics may be generated.^35,36^ This ease of derivatisation, coupled with their excellent NIR absorptivity, lead us to consider that porpholactones could be used as the starting point for developing an “all-in-one” phototheranostic.

**Fig. 1:**
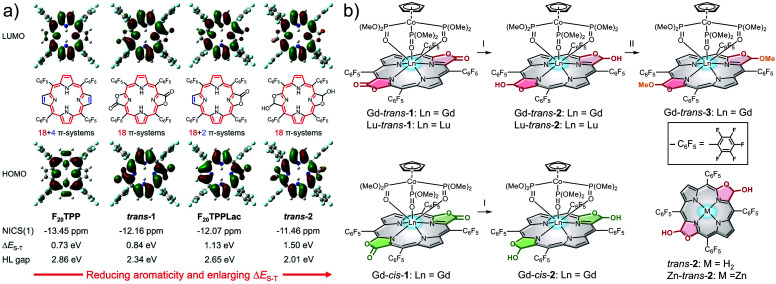
(a) Optimised geometries, frontier molecular orbitals, NICS(1) values and HOMO–LUMO (H–L) gaps from DFT calculations of porphyrin and porpholactone derivatives in the ground state (S_0_), as well as the adiabatic energy difference between the S_1_ and T_1_ states (Δ*E*_S–T_). Conjugated 18 π-electron pathways are highlighted in red, while cross-conjugated double bonds are shown in blue; the overall π-electron counts are also noted. (b) Structures of the porphodilactols and metalloporphodilactols considered in the present study. Conditions: (I) (1) DIBAL, THF, r.t; (2) H_2_O and (II) BF_3_·Et_2_O, MeOH, r.t.

Theoretical calculations were first performed on a series of porpholactone and porphyrin derivatives (F_20_TPP, *trans*-**1**, F_20_TPPLac and *trans*-**2**; Fig. 1a) using B3LYP/6-311G(d) to estimate the highest occupied molecular orbital (HOMO, H) and lowest unoccupied molecular orbital (LUMO, L) distributions and the Δ*E*_S–T_ values (see details in the ESI†). In addition, the aromatic character of these porphyrinoids was estimated by calculating the corresponding isotropic nucleus independent chemical shift (NICS(1)) values. As shown in Fig. 1a, the order of aromaticity is F_20_TPP > *trans*-**1** > F_20_TPPLac > *trans*-**2** as inferred from the NICS(1) calculations. The pyrrolic N–H proton shifts determined by ^1^H NMR spectroscopy proved consistent with this trend (Fig. S2†).

On the basis of our calculations and consistent with previous observations,^37^ the carbonyl units were found to be in conjugation with the macrocycle. As a result, reduction of the lactone unit of, *e.g.*, *trans*-**1** to its corresponding β-hydroxyl derivative, *trans*-**2**, leads to a strong decrease in the aromaticity.^38^ This reduction in aromaticity serves to narrow the H–L gap and red-shift the lowest energy absorption. On this basis *trans*-**2** was considered attractive as a potential phototheranostic. Unfortunately, however, the reductive conversion of *trans*-**1** to *trans*-**2** leads to an increase in the calculated Δ*E*_S–T_ gap from 0.84 to 1.50 eV. Such an increase was not expected to favour efficient ISC as needed for effective PDT.

To improve the PDT properties of *trans*-**2**, formation of various metal complexes was considered. The inclusion of metal ions into chromophores has previously been shown to enlarge the SOC matrix element and enhance the efficiency of ISC.^39^ Heavy metals, such as Au, Ir, and Pt, have large SOC constants (*ζ* > 3000 cm^−1^). This leads to fluorescence-based energy dissipation pathways being blocked. The net result is effective PDT behaviour coming at the cost of the optical features considered desirable for a phototheranostic.^39,40^ Therefore, lanthanide ions, Gd^3+^ and Lu^3+^ are often used to create porphyrinoid complexes since they have *ζ* values^39^ that are expected to preclude fluorescence quenching while enhancing the ISC efficiency.^41^ Based on these considerations, the Gd^3+^ and Lu^3+^ complexes of *trans*-**2** were prepared. While regioisomerically pure to the limits of our analysis, it is to be noted that these complexes are a mixture of stereoisomers. They were studied as such.

In brief, Gd^3+^ and Lu^3+^*trans*-5,10,15,20-tetrakis(pentafluorophenyl)porphodilactol (Ln-*trans*-**2**) were synthesised *via* the diisobutylaluminum hydride (DIBAL)-mediated reduction of the β-oxazolone units present in the corresponding metalloporphodilactone precursors (Fig. 1b).^42^ Similarly, Gd-*cis*-**2**, free base *trans*-**2**, and the corresponding zinc complex, Zn-*trans*-**2**, were synthesized using DIBAL and either Gd-*cis*-**1**, *trans*-**1**, and Zn-*trans*-**1** as starting materials, respectively (Fig. 1b and Scheme S1†). The methylated derivative, Gd-*trans*-**3**, was synthesised *via* BF_3_·Et_2_O catalysed methylation of the β-hydroxyl moiety of Gd-*trans*-**2** with MeOH.^43^ Detailed synthetic procedures and characterization data for all new compounds, including ^1^H NMR and high-resolution mass spectrometric (HRMS) analyses, can be found in the ESI (Fig. S24–S32†).

### Photophysical properties

**Fig. 2:**
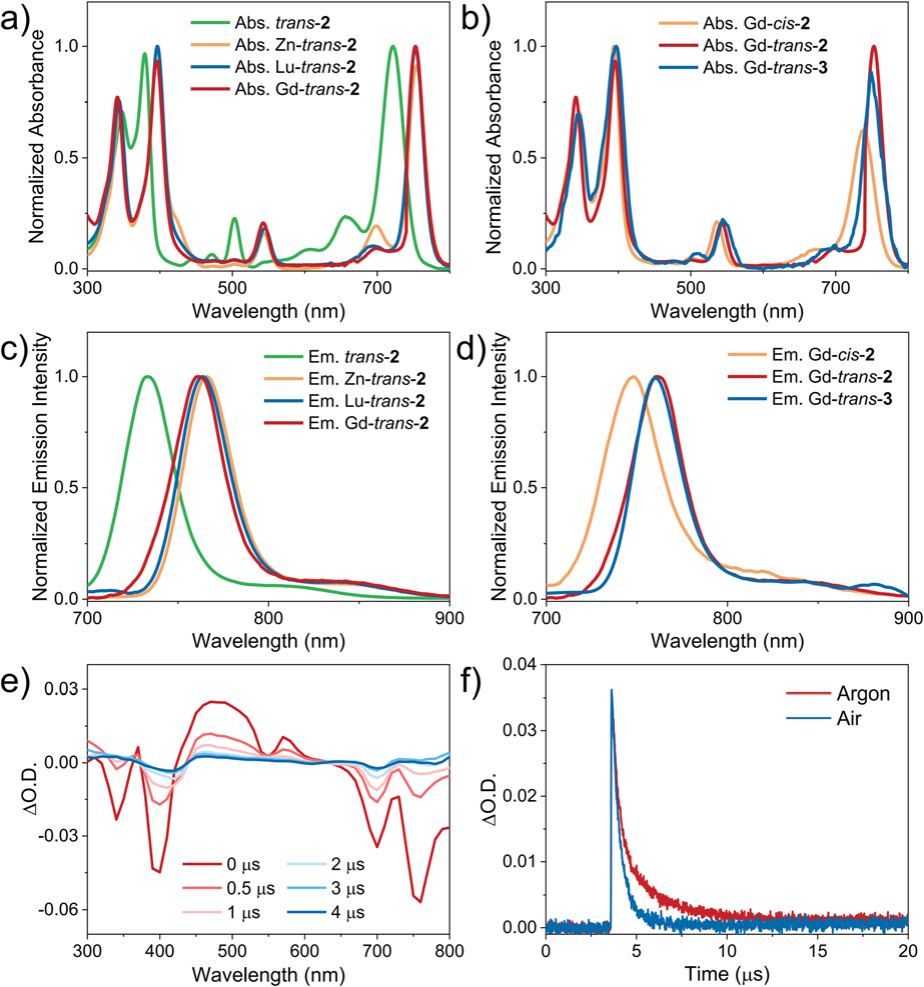
(a) and (b): Normalised (a) absorption and (b) emission spectra (*λ*_ex_ = 395 nm) of *trans*-**2**, and Zn-, Gd-, and Lu-*trans*-**2** (5 μM) in DCM at room temperature. (c) and (d) Normalised (c) absorption and (d) emission spectra (*λ*_ex_ = 395 nm) of Gd-*cis*-**2**, Gd-*trans*-**2** and Gd-*trans*-**3** (at a PS concentration chosen to give an absorbance of 0.1 at 760 nm (*A*_760nm_) in a 1 cm quartz cell) in DCM at room temperature. (e) Transient absorption difference spectra of Gd-*trans*-**2** measured at several delay times in deaerated toluene (*λ*_ex_ = 355 nm). (f) Decay traces of Gd-*trans*-**2** in deaerated (red) and air-saturated (blue) toluene. The intensity of the signal recorded under ambient atmospheric conditions is normalized to that seen in deaerated toluene.

The absorption and fluorescence emission spectra of the compounds included in the present study were recorded in dichloromethane (DCM) (Fig. 2). Corresponding photophysical data are included in Table 1. It was found that *trans*-**2** displayed bacteriochlorin-type absorption features, including split Soret bands at 300–400 nm, a weak Q_*x*_ band absorption at 500–550 nm, and an intense Q_*y*_ band at 650–750 nm. The corresponding Gd^3+^, Lu^3+^ and Zn^2+^ complexes displayed red-shifted (*ca.* 30 nm) Q_*y*_(0,0) bands (*λ*_max_ = 756 nm). Gd-*cis*-**2** displayed a blue-shifted (by 23 nm) Q_*y*_(0, 0) absorption band (*λ*_max_ = 738 nm) and a lower molar absorption coefficient *ε* = 9.8 × 10^4^ M^−1^ cm^−1^ relative to the *trans*-isomer (*λ*_max_ = 756 nm, *ε* = 1.5 × 10^5^ M^−1^ cm^−1^). This was believed to be the result of a regioisomeric effect seen previously in the case of *cis*/*trans*-**1**.^30^ The photophysical properties of Gd-*trans*-**2** and Gd-*trans*-**3** proved very similar (Fig. 2).

**Table 1:** Photophysical data for porphodilactol and metalloporphodilactol derivatives

Compound	Absorption^a^*λ*_max_/nm (lg *ε*/[M^−1^ cm^−1^])			Fluorescence^a^		Triplet^b^		*Φ*_Δ_ d (%)
	Soret bands	Q_*x*_(0, 0)	Q_*y*_(0, 0)	*λ*_max_/nm (*τ*_F_/ns)	*Φ*_FL_ (%)	*τ*_T_ (μs)	*k*_q_ c (M^−1^ s^−1^)	
*trans*-**2**	348 (4.95), 379 (5.08)	503(4.46)	722(5.10)	733 (2.5)	5.9	—	—	27
Zn-*trans*-**2**	332 (5.08), 387 (5.16)	534(4.54)	754(5.19)	766 (2.3)	4.2	—	—	32
Lu-*trans*-**2**	344 (5.01), 397 (5.14)	545(4.42)	753(5.13)	764 (1.2)	2.0	—	—	35
Gd-*cis*-**2**	346 (5.04), 394 (5.19)	536(4.52)	738(4.99)	748 (1.1)	1.5	—	—	—
Gd-*trans*-**2**	344 (5.05), 396 (5.17)	545(4.43)	753(5.17)	761 (1.1)	2.9	0.37(68%), 2.3(32%)	0.69 × 10^9^	37
Gd-*trans*-**3**	345 (4.99), 397 (5.15)	545(4.50)	749(5.10)	760 (1.4)	4.6	0.38(52%), 2.2(48%)	0.11 × 10^9^	33

a Determined in DCM at room temperature. *Φ*_FL_ was determined using ZnTPP (*Φ*_FL_ = 0.033, in toluene^44^) as the reference.

b Determined by transient absorption (TA) spectroscopy in toluene at room temperature (*λ*_ex_ = 355 nm).

c The oxygen quenching rate constant of the triplet excited state was calculated as *k*_q_ = (1/*τ*_T_ − 1/*τ*_T0_)[O_2_],^45^ where *τ*_T_ and *τ*_T0_ represent the triplet lifetimes in the absence and presence of oxygen, respectively.

d Determined in CHCl_3_ using H_2_TPP (*Φ*_Δ_ = 0.55, in CHCl_3_ (ref. 46)) as the reference.

Each compound associated with the present study, namely *trans*-**2**, and Zn-, Gd-, and Lu-*trans*-**2**, Gd-*cis*-**2**, and Gd-*trans*-**3**, was found to fluoresce in the ∼700–900 nm NIR region with decay lifetimes of 1.1–2.5 ns in DCM (Fig. 2 and Table 1). No phosphorescence was detected either in degassed solution or at 77 K. The fluorescence quantum yield of the metal-free ligand *trans*-**2** was 5.9%, which was the highest within the set. Upon photoexcitation (*λ*_ex_ = 395 nm) *trans*-**2** emits with maxima at 733 and 820 nm and is characterised by a fluorescence lifetime of 2.5 ns (Fig. S3†). In contrast, the corresponding metal complexes were found to display fluorescence emission features that were red-shifted by 30 nm relative to *trans*-**2** and quantum yields in the range of 2.0 to 4.2%. The increase in quantum yield values were seen to correlate with a decrease in the metal SOC constants (*ζ*), an increase in the Δ*E*_S–T_ gap and introduction of a β-hydroxyl group. As shown in Table 1, the larger SOC constants for the Gd^3+^ and Lu^3+^ ions (*ζ*_s_ = 1653 and 1151 cm^−1^, respectively) compared to Zn^2+^ (*ζ* = 390 cm^−1^)^17^ resulted in porphyrinoids with lower fluorescence quantum yields (*Φ*_FL_ 2.0 and 2.9%) relative to the Zn^2+^ complex (*Φ*_FL_ 4.2%). Traditionally, Lu^3+^ and Gd^3+^ porphyrin- or porphodilactone-based complexes emit phosphorescence rather than fluorescence.^48^ In contrast, the present *trans*-**2** species were found to be fluorescent. This contrasting emission behaviour serves to underscore the importance of the Δ*E*_S–T_ gap (a function of porphyrinoid ligand aromaticity) and the SOC constants of each metal ion. Notably, Gd-*trans*-**3** displayed an increased lifetime (1.4 ns) and an enhanced fluorescence emission quantum yield (*i.e.*, *Φ*_FL_ – 1.6-fold greater) relative to Gd-*trans*-**2**. This finding is rationalised in terms of high-energy O–H vibrations in Gd-*trans*-**2** that serve to quench slightly the excited singlet state.

### Evaluation of ROS generation and the photothermal effect

**Fig. 3:**
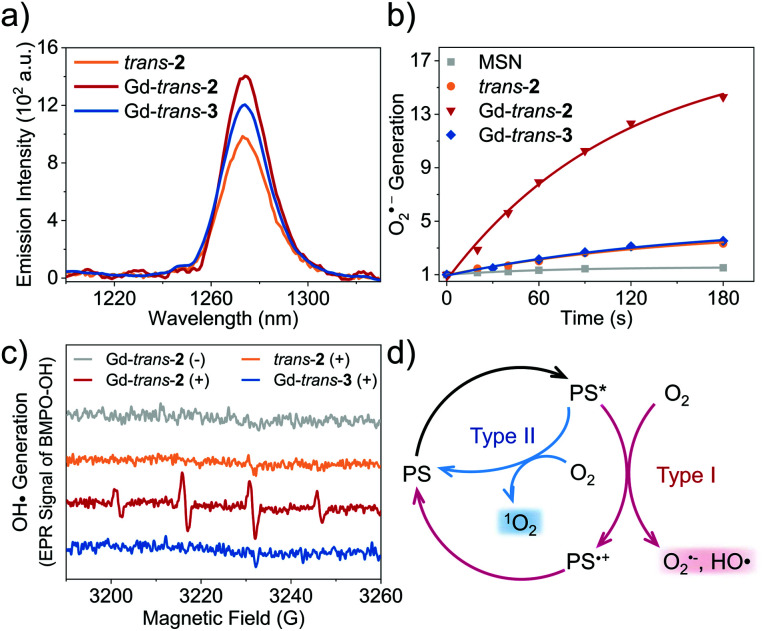
(a) NIR emission of ^1^O_2_ at 1270 nm induced by *trans*-**2**, Gd-*trans*-**2** and Gd-*trans*-**3** upon photo-irradiation at 760 nm (at a PS concentration such that *A*_760nm_ = 0.25) in CHCl_3_ under a normal ambient atmosphere. (b) Normalised time dependent O_2_˙^−^ generation promoted by irradiation of MSN-*trans*-**2** (orange), MSN-Gd-*trans*-**2** (red), and MSN-Gd-*trans*-**3** (blue) and blank (*i.e.*, PS-free) MSN (at the same concentration as the other groups, grey) detected using hydroethidine (DHE) as the fluorescent probe. (c) HO˙ generation promoted by irradiation, detected using EPR analysis with BMPO as the spin-trap. The experiments in (b) and (c) were carried out in aqueous solution at sample concentrations chosen such that *A*_760nm_ = 0.25 (photo-excitation at 760 nm, 7.5 mW cm^−2^). (d) Proposed mechanism for the Type I (red) and Type II (blue) photo-activation of molecular oxygen promoted by Ln-*trans*-**2**.

To determine the applicability of the present metal complexes as PDT photosensitisers (PSs), we first examined their ability to generate ^1^O_2_ upon light irradiation (760 nm, CHCl_3_). The ^1^O_2_ quantum yields (*Φ*_Δ_s) were determined through the emission of ^1^O_2_ at ∼1270 nm (^1^Δ_g_ → ^3^Σ_g_ transition of ^1^O_2_). Tetraphenylporphyrin (H_2_TPP, *Φ*_Δ_ = 0.55 in CHCl_3_ (ref. 46)) was used a reference.^47^ As shown in Table 1, Fig. 3a and S4,† each metal complex was shown to have an approximately 20–40% higher *Φ*_Δ_s than the free ligand (*trans*-**2**). This finding is consistent with the design expectation, namely that metal complexation facilitates population of the triplet excited state, which in turn is able to transfer energy to O_2_*via* a Type II pathway to produce ^1^O_2_. In previous research, we found that Gd-based porphodilactones have large HOMO–LUMO energy gaps, which results in excellent ^1^O_2_ production (up to 95% quantum yield).^48^ However, these prior systems lack the capacity to generate other ROS (*e.g.*, through a Type I pathway).

As noted above, Type I ROS production is highly desirable, particularly under hypoxic conditions. Therefore, we turned our attention towards testing whether other ROS, such as the superoxide anion, O_2_˙^−^ and the hydroxyl radical, HO˙, could be generated from *trans*-**2** and its complexes. This was done by using hydroethidine (DHE) as a fluorescent probe for O_2_˙^−^ (Fig. 3b),^49^ and 5-*tert*-butoxycarbonyl-5-methyl-1-pyrroline-*N*-oxide (BMPO) as a spin trap for HO˙ (Fig. 3c) with monitoring by means of electron paramagnetic resonance (EPR) spectroscopy.^50^ As these detection protocols required the use of aqueous media, we encapsulated each compound into mesoporous silica nanoparticles (MSN) (Scheme S2 and Fig. S4–S6†).^51^ This MSN strategy was shown to provide formulations that were well-dispersed in aqueous media, characterised by excellent biocompatibility, and photophysical properties that were basically unchanged relative to the free systems (Fig. S7†).

The introduction of the lactol unit was found to promote the production of Type I ROS. The O_2_˙^−^ quantum yields of MSN-Lu- and Gd-*trans*-**2** were calculated to be 23% and 21%, respectively (methylene blue was used a reference) (Fig. 3b).^52^ A remarkable 6- to 7-fold increase in O_2_˙^−^ generation was observed compared to MSN-Gd-*trans*-**3** or free base *trans*-**2** (3.6% and 3.1%). Similar results were obtained for HO˙ generation (Fig. 3c). Next, we used the ^1^O_2_ scavenger, sodium azide (NaN_3_) to exclude any potential conversion of ^1^O_2_ to other ROS, during these experiments. These studies revealed that the production of HO˙ is unaffected by and independent of ^1^O_2_ formation (Fig. S8†). This was taken as further evidence of ROS being produced *via* a Type I pathway.

It is important to appreciate that in addition to producing ROS *via* a Type I process, both MSN-Gd-*trans*-**2** and Gd-*trans*-**3** are able to promote the production of ^1^O_2_*via* a Type II pathway (*Φ*_Δ_s ∼ 30%, Fig. 3a). Previous studies that have sought to tune the ratio between Type I and Type II pathways have resulted in significant changes to the electronic structures as well as photophysical properties of the photosensitiser.^53–55^ This has made optimisation of various putative phototheranostics difficult. Gratifyingly, the lanthanide complexes of this study appear to fall in an appropriate “sweet spot”.

Similar photophysical properties were seen for MSN-Lu-*trans*-**2** and MSN-Gd-*trans*-**2**. Preliminary experiments revealed both Zn-*trans*-**2** and Lu-*trans*-**2** suffered from poor stability in solution (dichloromethane), whereas good stability was observed for the corresponding Gd^3+^ construct (Gd-*trans*-**2**) (Fig. S9†). Additionally, while not a focus of the present study, the paramagnetic Gd^3+^ centre present in MSN-Gd-*trans*-**2** might make this system of interest as a possible MRI contrast agent. Therefore, MSN-Gd-*trans*-**2** was chosen for further in-depth study with an emphasis being placed on understanding the role, if any, of the β-hydroxyl subunit.

Our working hypothesis is that the presence of the free β-hydroxyl unit accelerates the electron transfer processes critical to Type I reactions between ground-state oxygen and the Gd-*trans*-**2** excited state(s). To test this hypothesis, nanosecond transient absorption spectroscopic studies of both Gd-*trans*-**2** and its *O*-methylated derivative, Gd-*trans*-**3**, were carried out. Each compound was excited using a 355 nm laser in deaerated toluene. As shown in the difference spectra, the triplet state features include maxima at 370, 470, and 570 nm and ground-state bleaching of the Soret- and Q-band absorptions at 340, 400, 700 and 760 nm (Fig. 2e). The triplet state of Gd-*trans*-**2** was shown to decay *via* a double-exponential, with lifetimes of 0.37 μs (68%) and 2.3 μs (32%), respectively. Similar analyses were then carried out in the presence of air. Under these latter aerobic conditions, Gd-*trans*-**2** displayed a faster decay characterised by a double-exponential function (lifetimes = 0.35 μs (67%) and 0.75 μs (33%), respectively, Fig. 2f).

While not a proof, such a finding is consistent with an equilibrium between two distinct excited states where one (*ca.* 33%) is sensitive to oxygen (2.3 μs → 0.75 μs) and the other not. From these experiments, the oxygen quenching rate constant (*k*_q_) and quenching efficiency (*Φ*_q_) were estimated to be 0.69 × 10^9^ M^−1^ s^−1^ and 69%, respectively.^45^ Similar studies were carried out with Gd-*trans*-**3** yielding *k*_q_ and *Φ*_q_ values of 0.11 × 10^9^ M^−1^ s^−1^ and 31%, respectively (Fig. S10†). The difference in the decay kinetics for Gd-*trans*-**2** and Gd-*trans*-**3** provides support for the notion that the β-hydroxyl group plays a role in mediating the oxygen-induced deactivation of the triplet excited states. Previously, it has been shown that enzymes and their rationally designed synthetic models often incorporate hydroxyl groups into their structures to accelerate electron transfer processes *via* proton coupled electron transfer (PCET) mechanisms.^56^ On this basis, we suggest the role of the β-hydroxyl unit is to promote Type I ROS generation *via* PCET between the triplet excited state(s) of the metalloporphodilactol Gd-*trans*-**2** and molecular oxygen.

**Fig. 4:**
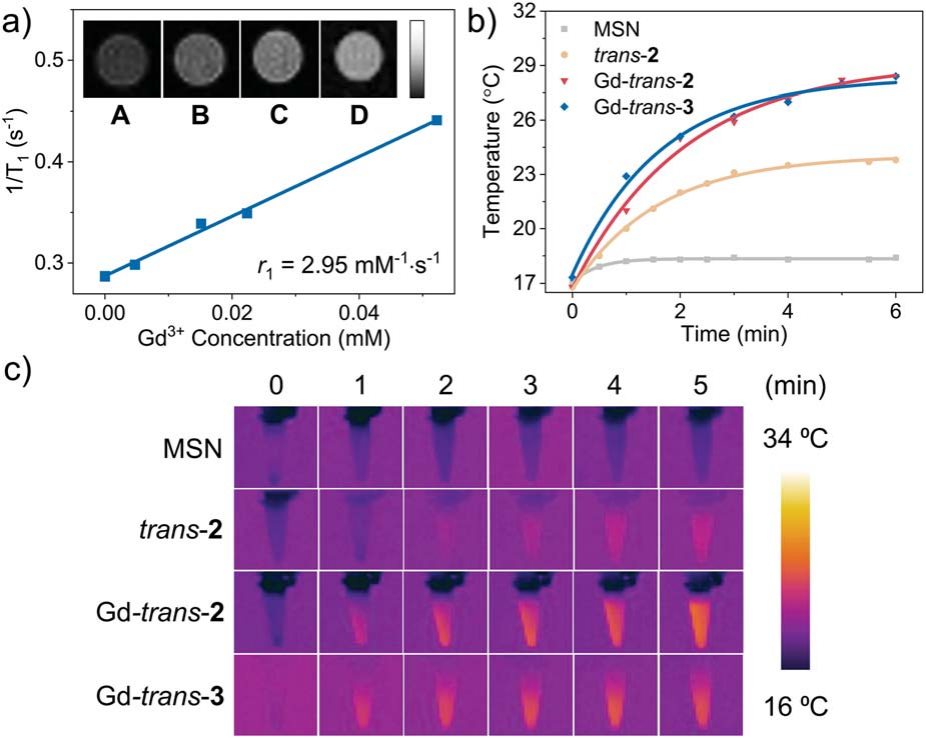
(a) *T*_1_ relaxation rate of MSN-Gd-*trans*-**2** in aqueous solution. Inset shows the *T*_1_ weighted MR images at different Gd-*trans*-**2** concentrations. (A–D) 0, 0.005, 0.02, 0.05 mM. (b) Photothermal effect of blank MSN, *trans*-**2**, Lu-*trans*-**2**, Gd-*trans*-**2** and Gd-*trans*-**3** produced upon photo-irradiation (760 nm, 100 mW cm^−2^, 5 min) at a sensitizer concentration chosen such that *A*_760nm_ = 1. (c) IR thermal images generated following photo-irradiation per the conditions in (b).

The paramagnetic properties of Gd^3+^ cations have been extensively exploited for the development of *T*_1_-contrast MRI agents.^57^ As a result, we determined the longitudinal relaxivity (*r*_1_) value for MSN-Gd-*trans*-**2**. As shown in Fig. 4a, *r*_1_ was measured to be 2.95 mM^−1^ s^−1^; moreover, the contrast in phantom MR images of MSN-Gd-*trans*-**2** as an aqueous dispersion increased with increasing concentration. In spite of these intriguing findings, a decision was made to focus the present study on exploring the phototheranostic potential of MSN-Gd-*trans*-**2**.

Recently, molecular systems with excited states that undergo effective nonradiative (NR) decay have been explored as photoacoustic imaging (PAI) and photothermal therapy (PTT) agents.^58,59^ Both MSN-Gd-*trans*-**2** and MSN-Gd-*trans*-**3** were found to be easily dispersed in water. Photo-irradiation (760 nm, 6 minutes, 100 mW cm^−2^) of the resulting solutions was found to increase the temperature by ∼12 °C with a photothermal conversion efficiency (*η*) of *ca.* 30% (Fig. 4b).^60^ No significant difference between the Gd-*trans*-**2** and Gd-*trans*-**3** MSN formulations was observed, reflecting energy dissipation pathways that are not affected by the β-hydroxyl unit. In comparison a smaller increase in the solution temperature was seen for the free ligand MSN-*trans*-**2** (7.0 °C, *η* = 23%). The better performance seen for the Gd^3+^ complexes is believed to reflect a heavy atom effect, which enhances nonradiative conversion thereby improving the photothermal performance.^61^ Lastly, as seen in Fig. 4c, IR thermal images were produced after laser irradiation (760 nm, 5 min, 100 mW cm^−2^), a result underscoring the potential of metalloporphodilactols, such as Gd-*trans*-**2** and Gd-*trans*-**3**, for IR photothermal imaging as well as PTT applications. Overall, these results provide support for the contention that Gd-*trans*-**2** and Gd-*trans*-**3** could serve as “all-in-one” phototheranostics capable of supporting NIR fluorescence/PA imaging and PTT/PDT-based therapies.

### *In vitro* experiments

**Fig. 5:**
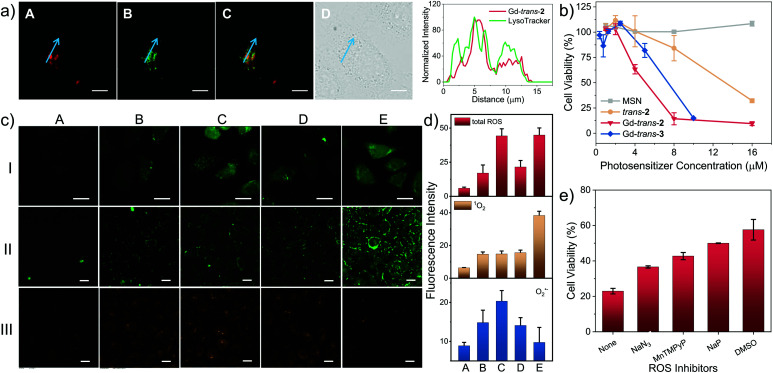
(a) Left: Fluorescence colocalization images of HeLa cells incubated with Gd-*trans*-**2** (5 μM Gd-*trans*-**2**) and LysoTracker® Green (75 nM). Column A–D: Gd-*trans*-**2** (*λ*_ex_ = 740 nm, *λ*_em_ = 776 nm long-pass), LysoTracker® Green (*λ*_ex_ = 488 nm, *λ*_em_ = 525/50 nm band-pass), merged image of A and B, and differential interference contrast (DIC) image. Right: the corresponding fluorescence profile analysis. (b) Photocytotoxicity of blank MSN (grey), *trans*-**2** (orange), Gd-*trans*-**2** (red) and Gd-*trans*-**3** (blue) against 4T1 cells under conditions of photo-irradiation (760 nm, 7.5 mW cm^−2^, 30 min), as determined using a CCK-8 assay. (c) *In vitro* detection of total ROS (lane I, green), ^1^O_2_ (lane II, green) and O_2_˙^−^ (lane III, orange) generation using H2DCFDA, an SOSG kit and DHE as the fluorescent probe, respectively. Column A–E shows the results of cells treated with PBS (+), *trans*-**2** (+), Gd-*trans*-**2** (+), Gd-*trans*-**3** (+) and Gd-*trans*-**1** (+), respectively. “+” and “−” denote with and without photo-irradiation respectively. Scale bar = 20 μm. (d) Quantitative analysis of cellular ROS generation based on the fluorescence intensity in (c). 20 cells were randomly selected for the quantification; the intensity is presented as the mean ± SD. (e) Photocytotoxicity of Gd-*trans*-**2** against 4T1 cells in the presence of NaN_3_ (10 mM), MnTPyP (100 μM), NaP (10 mM) and DMSO (0.28 M) as ROS scavengers, respectively (Gd-*trans*-**2** = 8 μM, 760 nm, 7.5 mW cm^−2^, 30 min).

To evaluate the potential of Gd-*trans*-**2** and Gd-*trans*-**3** to function as phototheranostics in biological settings, the “dark” toxicity and phototherapeutic efficacy of the corresponding MSN formulations were evaluated using a breast cancer 4T1 cell line with a Cell Counting Kit-8 (CCK-8). In the absence of light, a negligible cytotoxic effect was observed (Fig. S11†). In contrast, under conditions of photo-irradiation (760 nm, 7.5 mW cm^−2^, 30 min), a dose-dependent therapeutic effect was seen with MSN-Gd-*trans*-**2** outperforming both the free ligand MSN-*trans*-**2** and the *O*-methylated derivative MSN-Gd-*trans*-**3** as reflected in IC_50_ values of 4.1 ± 0.2, 13.4 ± 0.4 and 8.1 ± 0.4 μM, respectively (Fig. 5b). Similar studies were then carried out under lower oxygen tension. A decrease in photocytotoxicity was observed as expected for a process wherein reaction with oxygen contributes to the overall phototoxicity (Fig. S12†).

The cellular localisation of a therapeutic often determines its efficacy and selectivity.^24^ Therefore the localisation of MSN-Gd-*trans*-**2** and several control systems within living cells was probed using confocal fluorescence microscopy. As shown in Fig. 5a, an intense fluorescence signal from MSN-Gd-*trans*-**2** was observed. Co-localisation experiments using commercial LysoTracker® Green (Pearson's coefficient = 0.74) provided support for the accumulation of MSN-Gd-*trans*-**2** in lysosomes. Both MSN-*trans*-**2**, and MSN-Gd-*trans*-**3** were shown to have similar Pearson's coefficients > 0.70 with LysoTracker® Green (Fig. S13†).

Subsequently, the light-mediated generation of various ROS, namely total ROS, ^1^O_2_ and O_2_˙^−^, was determined *in vitro* by confocal fluorescence microscopy using 2′,7′-dichlorodihydrofluorescein diacetate (H2DCFDA), a commercially available Singlet Oxygen Sensor Green (SOSG) assay, and DHE as fluorescent markers for these ROS, respectively. Upon photo-irradiation (760 nm, 7.5 mW cm^−2^, 30 min), 4T1 cells treated with MSN-Gd-*trans*-**2** (Fig. 5c, column C) produced obvious green (lane I), green (lane II) and orange (lane III) fluorescence responses, respectively. This was taken as evidence for the production of each corresponding ROS. This inference was further confirmed by various control experiments where a minimal increase in the fluorescent intensity was observed (*cf.*, *e.g.*, column A: photo-irradiation in the absence of Gd-*trans*-**2**).

A sharp contrast was seen between MSN-Gd-*trans*-**2** and MSN-Gd-*trans*-**1** in terms of their respective Type I and II ROS production. For instance, only a strong fluorescence intensity was observed for ^1^O_2_ production mediated by MSN-Gd-*trans*-**1** (Fig. 5c, Column E). In contrast, MSN-Gd-*trans*-**3** and MSN-*trans*-**2** displayed a much smaller increase in the fluorescence ascribed to the production of O_2_˙^−^ but produced a ^1^O_2_ signal comparable to that of MSN-Gd-*trans*-**2** (Columns D and B). For each compound, we estimated the generation of total ROS, ^1^O_2_ and O_2_˙^−^ from the ratio of fluorescence intensity of the putative photosensitiser *vs.* the corresponding blank (Column A). As shown in Fig. 5d (quantified using the images in Fig. 5c), similar ^1^O_2_ generation was observed for all compounds. However, overall, a greater level of total intracellular ROS generation was seen for MSN-Gd-*trans*-**2** relative to the other agents considered in this study. This finding thus mirrors what was seen in the predicative aqueous solution studies discussed above (Fig. 3, *supra*).

To confirm that ROS generation is essential for the phototherapeutic efficacy seen in the case of MSN-Gd-*trans*-**2**, we used sodium azide (NaN_3_), Mn(ii)-*tetrakis*-(4-*N*-methylpyridiniumyl)porphyrin (MnTPyP), dimethyl sulfoxide (DMSO) or sodium pyruvate (NaP) as ROS scavengers for ^1^O_2_, O_2_˙^−^, HO˙ and H_2_O_2_ species, respectively. These ROS scavengers were found to inhibit only partially the photocytotoxicity (Fig. 5e). This was taken as evidence that along with both Type I and II PDT, PTT effects contribute to the overall phototherapeutic efficacy seen for MSN-Gd-*trans*-**2**.

Further and more quantitative analyses of the therapeutic effect of MSN-Gd-*trans*-**2** were made *via* flow cytometry using a commercially available Annexin V Apoptosis Detection Kit. A relatively high percentage of apoptotic cells, *ca.* 40–50%, was seen after photo-irradiation (4 μM, 760 nm, 7.5 mW cm^−2^, 30 min), whereas fewer than 1% of apoptotic cells were observed under “dark” conditions or in the absence of MSN-Gd-*trans*-**2** (Fig. S14†). The activity of the apoptosis related biomarkers caspase-3 and poly ADP-ribose polymerase (PARP) were determined by western blot (Fig. S15†). The ratio of cleaved caspase-3 and PARP to β-actin increased by 1.8 and 4.7-fold in comparison to that of the dark control group (*p* < 0.01). These results are interpreted in terms of the induction of an apoptosis pathway during MSN-Gd-*trans*-**2**-mediated phototherapy.

### *In vivo* experiments

**Fig. 6:**
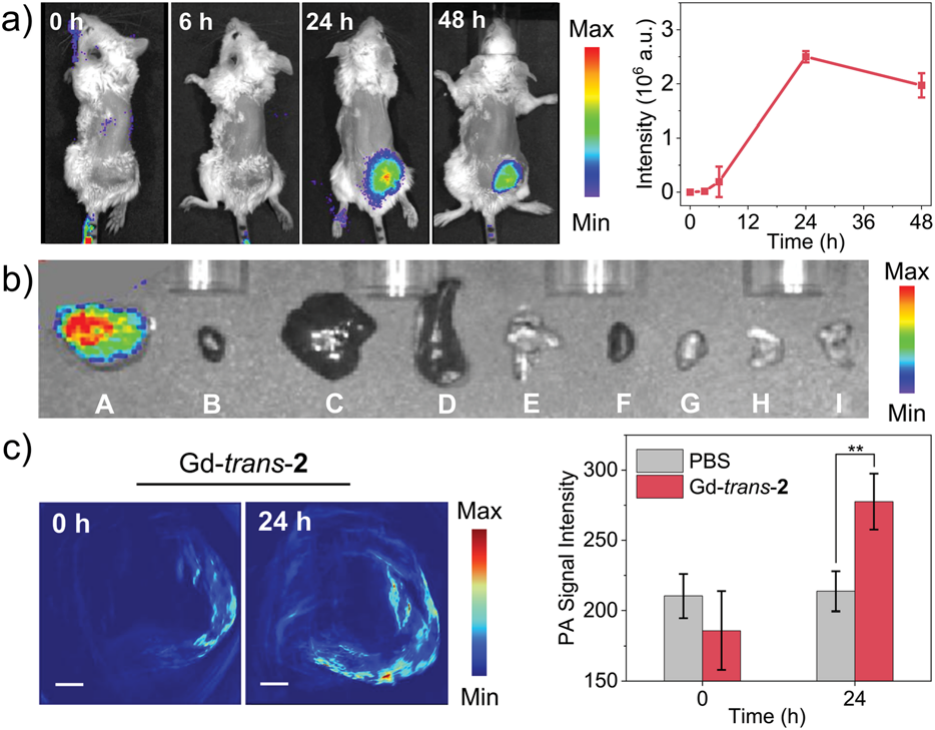
(a) *In vivo* NIR fluorescence imaging (left) and signal intensity changes in the tumour region (right, *n* = 3, mean ± SD) of 4T1-tumour-bearing mice after treatment with MSN-Gd-*trans*-**2** (tail vein injection, 1 mg kg^−1^; *λ*_ex_ = 740 nm, *λ*_em_ = 780 nm). (b) *Ex vivo* fluorescence imaging. (A–I) Tumour, heart, liver, spleen, lung, kidney, pancreas, stomach, and intestine. (c) PA imaging of 4T1-tumour-bearing mice before and 24 h after treatment with MSN-Gd-*trans*-**2** (left) and signal intensity changes in the tumour region (right, *n* = 3, mean ± SD, **p < 0.01). Tail vein injection, 1 mg kg^−1^ MSN-Gd-*trans*-**2**, *λ*_ex_ = 760 nm.

We next turned our attention to evaluating the potential of MSN-Gd-*trans*-**2** to serve as a phototheranostic *in vivo*. Recently, increasing attention has been devoted to multimodal imaging.^62,63^ Therefore, MSN-Gd-*trans*-**2** was first evaluated for its ability to be used as multimodal imaging agent *in vivo*. This would generate the information required for tumour diagnosis and identification of the optimal time point post-injection to carry out light-based therapy. NIR fluorescence imaging was performed by recording the fluorescence intensity (780 nm) at various time points after MSN-Gd-*trans*-**2** was administered *via* intravenous injection (1 mg kg^−1^, tail vein) in 4T1-tumour-bearing mice (Fig. 6a). A remarkable increase in the fluorescence intensity was observed at the tumour. A maximum value was seen *ca.* 24 h post-injection, as would be expected for an agent that accumulates gradually at the tumour site. To support this observation, the biodistribution of MSN-Gd-*trans*-**2** was analysed 24 h post-injection by means of *ex vivo* NIR fluorescence imaging. As can be seen from an inspection of Fig. 6b, the heart, lung, liver, spleen and kidney produced weak fluorescence signals, whereas a high fluorescence intensity was seen for the tumour. Similar results NIR imaging results were obtained in the case of MSN-Gd-*trans*-**3** and MSN-*trans*-**2**. This observation was supported by ICP-MS analyses; *cf.* Fig. S16–S18.†

We next examined the capability of MSN-Gd-*trans*-**2** to produce an *in vivo* PA signal in 4T1-tumour-bearing mice. As shown in Fig. 6c and S19,† the PA signal was shown to be enhanced in a statistically significant fashion 24 h post-injection compared to pre-injection. In contrast, the control group (PBS only), showed no apparent change in the PA signal (Fig. S20†). These findings provide a complement to the fluorescence data discussed above and support the conclusion that MSN-Gd-*trans*-**2** localises well in tumours. Considered in concert with the other imaging studies, these results also serve to underscore the fact that MSN-Gd-*trans*-**2** could have a role to play in multimodal tumour imaging.

**Fig. 7:**
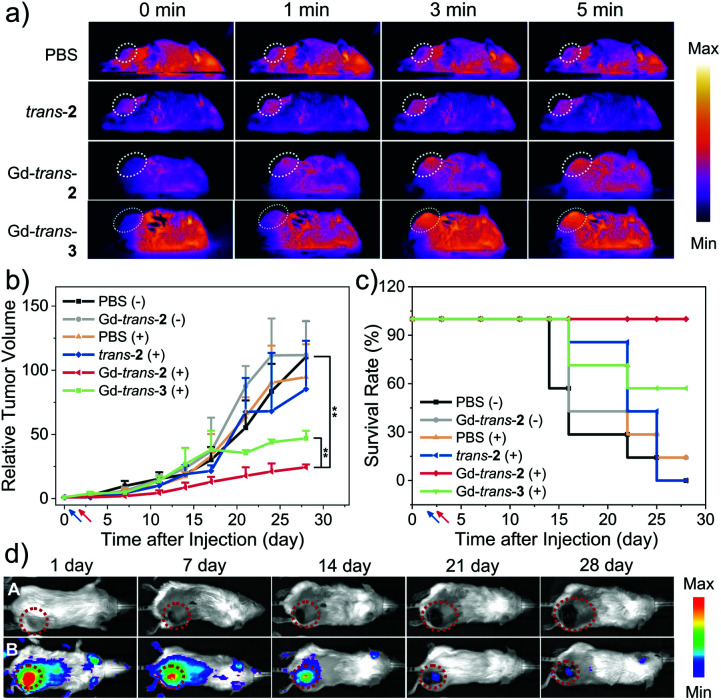
*In vivo* combination therapy. (a) IR thermal images of 4T1-tumour-bearing mice subject to laser irradiation 24 h post-injection of PBS, MSN-*trans*-**2**, MSN-Gd-*trans*-**2** or MSN-Gd-*trans*-**3** (1 mg kg^−1^; *λ*_ex_ = 760 nm, 100 mW cm^−2^, 5 min) (b) tumour growth profiles for different mice groups (**p < 0.01). (c) Standard Kaplan–Meier curves for different mice groups following treatment. Blue and red arrow represents the injection (0) and irradiation (1^st^) day, respectively; “+” and “−” denote with and without photo-irradiation (*λ*_ex_ = 760 nm, 100 mW cm^−2^, 5 min), respectively. (d) *In vivo* NIR fluorescence images recorded on days 1, 7, 14, 21, and 28 day post-injection. Column A and B reflect treatment with PBS (Group 3) and MSN-Gd-*trans*-**2** (Group 7), respectively. Animals were injected intravenously once on day 0, tail vein; *λ*_ex_ = 740 nm, *λ*_em_ = 780 nm.

The *in vivo* phototherapeutic efficacy of MSN-Gd-*trans*-**2** was then explored using a 4T1-tumour bearing mouse model. As a first step in this evaluation, IR thermal images at the tumour site were recorded following treatment with MSN-Gd-*trans*-**2**, MSN-*trans*-**2**, and MSN-Gd-*trans*-**3**, respectively, under conditions of photo-irradiation for 0, 1, 3, 5 min (1 mg kg^−1^ PS, 760 nm, 100 mW cm^−2^; Fig. 7a). A rapid temperature rise (22–32 °C) at the tumour site was seen for MSN-Gd-*trans*-**2**, MSN-*trans*-**2**, and MSN-Gd-*trans*-**3** (Fig. S21†). In contrast, a negligible temperature (Δ*T* < 1 °C) rise was observed in the case of the PBS control group and only modest photothermal conversion efficiency was seen for the free ligand MSN-*trans*-**2** (Δ*T* = 4 °C). Again, these findings serve to highlight the importance of metal complexation for achieving a robust PTT effect.

The anti-tumour PDT/PTT efficacy of each PS was then evaluated in 4T1 tumour-bearing mice. A total of 42 mice were used. They were divided into 6 groups: (1) PBS and dark, (2) MSN-Gd-*trans*-**2** and dark, (3) PBS + light, (4) MSN-*trans*-**2** + light, (5) MSN-Gd-*trans*-**2** + light, (6) MSN-Gd-*trans*-**3** + light. Based on the localisation studies presented in Fig. 6, the animals in Group 3–6 were subjected to photo-irradiation at the tumour site 24 h post-injection (1 mg kg^−1^ PS, 760 nm laser, 100 mW cm^−2^, 5 min). The size of the tumours and body weights of the mice in each group were monitored twice weekly. As shown in Fig. 7b, rapid tumour growth was observed for the PBS-treated-group, as well as the groups treated with one of the test photosensitisers but without subjecting to photo-irradiation. In contrast, under conditions of photo-irradiation MSN-Gd-*trans*-**2** was shown effective for the inhibition of tumour growth. In the case of MSN-*trans*-**2**, mice treated with light were found to have the tumour growth partially inhibited, particularly for the first 1–2 weeks; however, rapid tumour regrowth was subsequently observed. Suppression of tumour growth was seen for the group treated with MSN-Gd-*trans*-**3** and subject to photo-irradiation, although the benefit was less than that seen in the case of MSN-Gd-*trans*-**2** (Fig. 7c). These results thus recapitulate the relative efficacy for these two species seen *in vitro* (Fig. 5, *supra*). Since MSN-Gd-*trans*-**2** and MSN-Gd-*trans*-**3** are characterized by similar *Φ*_Δ_ values and give rise to similar PTT effects, we conclude that a Type I PDT effect plays an important role in mediating the overall phototherapeutic efficacy.

No evidence of body weight loss or other abnormalities was seen in the case of all groups, leading us to conclude that the drug-loaded MSN formulations may be subject to minimal side effects (Fig. S22†). In addition, the fluorescence signal gradually diminished over time, which may reflect the eventual clearance of MSN-Gd-*trans*-**2** or its metabolism to less-emissive species (Fig. 7d and S22†). Therefore, in order to understand further the therapeutic effect and evaluate any potential toxicity of each PSs, the tumour tissues and major organs of each mouse group (heart, liver, spleen, lung and kidney) were isolated and subject to hematoxylin and eosin (H&E) staining (Fig. S23†). Notably, evidence of necrosis and apoptosis was observed in tumours treated with a combination of PS and laser irradiation, while those in the PBS or dark groups showed little change. Slices of organs revealed large and spindle shape nuclei in the case of all groups, leading us to conclude that treatment with MSN-Gd-*trans*-**2** engenders no obvious toxicity in the major organs.

As a test of whether the diagnostic components of phototheranostic MSN-Gd-*trans*-**2** might allow the therapeutic outcome of PTT/PDT-based treatments to be monitored, *in vivo* fluorescence images were obtained on days 1, 7, 14, 21, and 28 following a single injection on day 0 of MSN-Gd-*trans*-**2**. Similar monitoring was carried out in the case of a control injection with PBS. As shown in Fig. 7d, over the course of the phototherapy process, the NIR fluorescence signal gradually decreased (*λ*_ex_ = 740 nm, *λ*_em_ = 780 nm). On this basis we propose that MSN-Gd-*trans*-**2** acts as a stand-alone phototheranostic that shows promise of both treating and monitoring anti-tumour effects *in vivo*. It is appreciated, however, that optimisation of the treatment protocol, such as exploring the use of multiple injections spread out of multiple days, may be necessary to maximize the diagnostic and therapeutic utility of MSN-Gd-*trans*-**2**.

## Conclusions

Porphodilactol derivatives and their corresponding metal complexes have been explored as “all-in-one” phototheranostics. To create an effective system, namely MSN-Gd-*trans*-**2**, we felt it was important to balance the inherent photophysical properties with the ISC efficiency. Theoretical calculations, combined with photophysical measurements, revealed that a decrease in aromaticity serves to reduce the HOMO–LUMO energy gap and cause a red-shift in the absorption maximum. Unfortunately, this decrease in aromaticity leads to an increase in Δ*E*_S–T_ gap, which reduces the overall ISC efficiency. However, this latter limitation may be overcome by means of appropriate metal complexation. In the case of the porphodilactols of the present study, this could be achieved by formation of a Lu^3+^ or Gd^3+^ complex. The latter system displayed slightly more favourable photophysical features and proved more stable and was thus studied in depth. It was found that free β-hydroxyl units were essential for promoting Type I ROS production, a desired feature for the development of effective PDT-based agents capable of operating at lower oxygen tensions. A biological evaluation of the metalloporphodilactol Gd-*trans*-**2** as a phototheranostic was carried out after encapsulation in mesoporous silica nanoparticles. The resulting construct displayed excellent fluorescence and photoacoustic imaging capabilities. It also showed good PTT/PDT efficiency both *in vitro* and *in vivo* in 4T1-tumour-bearing mice. We thus propose that MSN-Gd-*trans*-**2** warrants further study as an “all-in-one” phototheranostic. Indeed, efforts are underway to fine-tune the administration regimes so as to exploit fully the therapeutic and diagnostic potential of this and other metalloporphodilactols.

## Ethical statement

All the animal experiments were performed in strict accordance with the National Institutes of Health Guide for the Care and Use of Laboratory Animals and were approved by the Institutional Animal Care and Use Committee of the Peking University First Hospital (Beijing, China).

## Author contributions

J.-L. Z, J. L. S., L. K., H. S., B.-W. W., and S. G. supervised the work. Z.-S. Y., C. L., Y. X. and Y. W. conducted the experiments. Y. Y. performed theoretical calculations. Z.-S. Y., Y. Y., A. C. S., J. L. S., and J.-L. Z wrote and revised the manuscript. All authors have been involved in checking the data, and have given approval to the final version of the manuscript.

## Conflicts of interest

There are no conflicts to declare.

## Supplementary Material

SC-011-D0SC03368E-s001
