# Supplementary material for: Rational design of an “all-in-one” phototheranostic
Source: Chem Sci. 2020 Jul 21;11(31):8204–13. doi: 10.1039/d0sc03368e (PMC8163340; doi:10.1039/d0sc03368e)
Supplement: SC-011-D0SC03368E-s001 [file SC-011-D0SC03368E-s001.pdf]

Electronic Supplementary Material (ESI) for Chemical Science.
This journal is © The Royal Society of Chemistry 2020

Supporting Information for:

Rational design of an "all-in-one" phototheranostic

Zi-Shu Yang,[a] Yuhang Yao,[a] Adam C. Sedgwick,[b] Cuicui Li,[c] Ye Xia,[d] Yan Wang,[a] Lei Kang*,[c] Hongmei Su,[d] Bing-Wu Wang,[a] Song Gao,[a, e] Jonathan L. Sessler*[b] and Jun-Long Zhang*[a]

[a] Beijing National Laboratory for Molecular Sciences, State Key Laboratory of Rare Earth Materials Chemistry and Applications, College of Chemistry and Molecular Engineering, Peking University, Beijing 100871, People's Republic of China

[b] Department of Chemistry, The University of Texas at Austin, 105 East 24th Street-A5300, Austin, TX 78712-1224, United States

[c] Department of Nuclear Medicine, Peking University First Hospital, Beijing 100034, People's Republic of China

[d] College of Chemistry, Beijing Normal University, Beijing 100875, People's Republic of China

[e] School of Chemistry and Chemical Engineering, South China University of Technology, Guangzhou, 510640 People's Republic of China

Email: zhangjunlong@pku.edu.cn; sessler@cm.utexas.edu; kanglei@bjmu.edu.cn

Table of Contents

1. **Experimental procedures** S4

Materials and general methods S4

Theoretical calculations S4

Synthesis of lanthanide porphodilactols S5

Synthesis of *trans*-2 and Zn-*trans*-2 .... S5

Synthesis of Gd-*trans*-3 .... S6

Preparation of porphyrinoid loaded mesoporous silica nanoparticles (MSN-PS) S6

Characterization of the loading capacity of PS in MSN-PS Nanoparticles S7

Detection of reactive oxygen species (ROS) S7

Measurement of the photothermal effect S8

Cell culture studies S9

***In vitro* cell imaging** S9

***In vitro* cytotoxicity studies** S9

Determining ROS generation levels in vitro S10

Apoptosis analyses S11

***In vivo* imaging experiments** S12

***In vivo* phototoxicity experiments** S12

Statistical analyses S13

2. **Additional Figures, Spectra and Tables** S13

Table S1 N-H ^{1}H NMR signals, and calculated $\Delta E_{S\text{-}T}$, HLgap, and NICS values of PS S13

Fig. S1 Relationship between calculated ΔES-T and NICS(1) values of PS. S14

Fig. S2 Relationship between the chemical shifts of the inner N–H proton signals and the calculated NICS(1) values of PS. S14

Fig. S3 Fluorescence decay curves of PS. S14

Fig. S4 The transmission electron microscopy (TEM) image of PS-MSN. S15

Fig. S5 The size distribution and DPI of PS-MSN. S15

Fig. S6 Dynamic light scattering (DLS) of MSN-Gd-*trans*-**2**. S15

Fig. S7 UV-Vis absorption of PS-MSN in water. S16

Fig. S8 HO• generation of Gd-*trans*-**2** promoted by irradiation in the presence of NaN_3. ... S16

Fig. S9 Stability of metal porphodilactols in DCM determined by UV-Vis absorption. S16

Fig. S10 Transient absorption spectra and kinetic trace of Gd-*trans*-**3** in toluene. S17

Fig. S11 Dark cytotoxicity of PS against 4T1 cells. S18

Fig. S12 Photocytotoxicity of Gd-*trans*-**2** against 4T1 cells under hypoxia. S18

Fig. S13 Fluorescence colocalisation images of Gd-*trans*-**3** in HeLa cells. S19

Fig. S14 Flow cytometry analysis of 4T1 cells treated with Gd-*trans*-**2** using an Annexin V-FITC/PI apoptosis kit. S19

Fig. S15 Western blotting for PARP and Caspase 3 with treatment of Gd-*trans*-**2**. S20

Fig. S16 *In vivo* NIR fluorescence imaging of 4T1-tumour-bearing mice treated with PS. ... S20

Fig. S17 *Ex vivo* fluorescence imaging 24 h post-injection of PS. S21

Fig. S18 Distribution of Gd^{3+} complexes in the tumour and main organs determined by ICP-MS. S21

Fig. S19 *In vivo* PA imaging of 4T1-tumour-bearing mice treated with PS. S21
Fig. S20 Temperature change of tumour surface of 4T1-tumour-bearing mice subject to laser irradiation at different time points. S21
Fig. S21 Relative body weights of mice during treatment of photoherapy. S22
Fig. S22 *In vivo* NIR fluorescence images recorded post-injection of Gd-*trans*-**2**. S22
Fig. S23 H&E staining images of tumour and primary organs after treatment of phototherapy. S23
Fig. S24 ^{1}H NMR spectrum of *trans*-**2**. S24
Fig. S25 ^{1}H NMR spectrum of Zn-*trans*-**2**. S24
Fig. S26 ^{1}H NMR spectrum of Lu-*trans*-**2**. S25
Fig. S27 HR-MS spectrum (ESI^+) of *trans*-**2**. S25
Fig. S28 HR-MS spectrum (ESI^+) of Zn-*trans*-**2**. S26
Fig. S29 HR-MS spectrum (ESI^+) of Lu-*trans*-**2**. S26
Fig. S30 HR-MS spectrum (ESI^+) of Gd-*trans*-**2**. S27
Fig. S31 HR-MS spectrum (ESI^+) of Gd-*cis*-**2**. S27
Fig. S32 HR-MS spectrum (ESI^+) of Gd-*trans*-**3**. S28
Table S2 F_{20}TPP S_0 optimised geometry. S28
Table S3 F_{20}TPP S_1 optimised geometry. S30
Table S4 F_{20}TPP T_1 optimised geometry. S33
Table S5 F_{20}TPPLac S_0 optimised geometry. S35
Table S6 F_{20}TPPLac S_1 optimised geometry. S37
Table S7 F_{20}TPPLac T_1 optimised geometry. S39
Table S8 *trans*-**1** S_0 optimised geometry. S42
Table S9 *trans*-**1** S_1 optimised geometry. S44
Table S10 *trans*-**1** T_1 optimised geometry. S46
Table S11 *trans*-**2** S_0 optimised geometry. S48
Table S12 *trans*-**2** S_1 optimised geometry. S51
Table S13 *trans*-**2** T_1 optimised geometry. S53
3. References S56

1. Experimental procedures

Materials and general methods

All reagents were purchased from commercial suppliers and used as received unless otherwise indicated. Anhydrous methanol (MeOH) were purchased from J&K Scientific and used as received. Anhydrous tetrahydrofuran (THF) was distilled from sodium (Na), and anhydrous dichloromethane (DCM) was distilled from calcium hydride (CaH_2). H_2O was obtained from Milli-Q Integral. ^1H NMR spectra were recorded using a 400 MHz Bruker spectrometer with tetramethylsilane (TMS, $\delta = 0.00$ ppm) as the standard. ESI mass spectra were recorded using a Bruker APEX IV FTICR mass spectrometer. The size and morphology of porphyrinoid-loaded MSN (PS-MSN) nanoparticles were determined using a JEOL JEM-2100F field-emission high resolution transmission electron microscope operated at 200 kV. UV-Vis spectra were recorded using a Agilent 8453 UV/Vis spectrometer equipped with an Agilent 89090A thermostat (± 0.1 °C). The fluorescence emission spectra and luminescence lifetimes were recorded using an Edinburgh Analytical Instruments FLS980 lifetime and steady-state spectrometer (450 W Xe lamp/microsecond flash lamp) equipped with a PMT R928 detector. A HAMAMATSU R5509-73 PMT with a C9940-02 cooler was used for the NIR fluorescence emission studies. Nanosecond time-resolved transient absorption spectra were measured using a flash photolysis setup Edinburgh LP920 spectrometer combined with a Nd: YAG laser. The sample was excited by a 355-nm laser pulse (1 Hz; 10 mJ per pulse) using a 450-W pulsed xenon lamp. Electron paramagnetic resonance (EPR) spectra were collected using a JEOL FA-200 Spectrometer. Photothermal (PTT) effects were determined using a OMEGA HH800A thermometer. Infrared (IR) imaging was performed using a FLIR E40 thermal imaging camera. Magnetic resonance imaging (MRI) was performed with a Bruker BioSpec70/20USR magnetic resonance instrument. Confocal fluorescence microscopy and fluorescence lifetime imaging of live cells was performed using an ISS Alba5 FLIM/FFS confocal system equipped with a Nikon TE2000 inverted microscope, and a 405 nm laser, a 488 nm laser and a YSL supercontinuum laser (450-1000 nm). Western blotting analyses were carried out using a Bio-Rad ChemiDoc Touch imaging system. Flow cytometry was performed with a Becton, Dickinson and Company BD FACSVerse flow cytometer. *In vivo* fluorescence imaging was achieved using a Xenogen IVIS spectrum. *In vivo* photoacoustic imaging (PAI) was carried out using an iTheraMedical MSOT Invision 128 optoacoustic imaging platform.

Theoretical calculations

Geometry optimisations for the ground (S_0) and excited states (S_1 and T_1) followed by harmonic vibrational frequencies calculations with the hybrid density functional, B3LYP[1, 2], were carried out using the program package Gaussian 09 (Revision E.01).[3] The 6-311G(d) basis set[4, 5] was used for

all atoms. Frequency calculations were performed on optimized structures to identify minimum energy structures in the absence of imaginary frequencies. The 6-31+G(d) basis set was employed for the NICS[6, 7] calculations.

Synthesis of lanthanide porphodilactols

General procedure. Porphodilactol and metalloporphodilactols were synthesized following previous literature protocols with slight modifications.[8] In brief, the starting porphodilactone/metalloporphodilactone (**1**/M-**1**) (0.05 mmol) was dissolved in dry THF (5 mL) and the reaction mixture cooled to – 78 °C. Excess DIBAL (0.1 M in hexanes, 5 equiv.) was then added dropwise to the solution and the reaction mixture stirred at room temperature for 1 h. The reaction was then quenched with H_2O. Volatiles were removed under reduced pressure and the crude mixture was purified *via* silica gel column chromatography using petroleum ether (PE)/ethyl acetate (EA) (v/v = 5:1) as the eluent. This afforded the desired porphodilactol/metalloporphodilactol. All compounds were obtained as red powders.

Synthesis of Lu-*trans*-2. General procedure starting with Lu-*trans*-**1**. Yield: 80%. ^{1}H NMR (400 MHz, $CHCl_3$-*d*): δ 8.02 (d, J = 4.5 Hz, 2H), 7.95 (d, J = 4.5 Hz, 2H), 7.68 (d, J = 8.9 Hz, 2H), 4.47 (s, 5H), 4.15 (d, J = 9.0 Hz, 2H), 2.77 (dd, J = 7.2, 3.6 Hz, 18H). HR-MS (ESI$^+$) *m/z* [M+H]$^+$: Calcd for $C_{53}H_{32}CoF_{20}LuN_4O_{13}P_3^+$ 1638.95975; found: 1638.95527. UV/Vis (CH_2Cl_2, 25 °C): λ_{max} (nm) (log ε): 341(5.01), 396(5.14), 503(3.77), 543(4.42), 697(4.15), 753(5.16).

Synthesis of Gd-*trans*-2. General procedure starting with Gd-*trans*-**1**. Yield: 85%. HR-MS (ESI$^+$) *m/z* [M+H]$^+$: Calcd for $C_{53}H_{32}CoF_{20}LGdN_4O_{13}P_3^+$ 1621.94445; found: 1621.94925. UV/Vis (CH_2Cl_2, 25°C): λ_{max} (nm) (log ε): 344(5.05), 397(5.17), 503(3.79), 545(4.43), 694(4.18), 753(5.17).

Synthesis of Gd-*cis*-2. General procedure starting with Gd-*cis*-**1**. Yield: 77%. HR-MS (ESI$^+$) *m/z* [M+H]$^+$: Calcd for $C_{53}H_{32}CoF_{20}LGdN_4O_{13}P_3^+$ 1621.94445; found: 1621.97887. UV/Vis (CH_2Cl_2, 25 °C): λ_{max} (nm) (log ε): 346 (5.04), 394 (5.19), 501(3.90), 536(4.52), 672(4.10), 738(4.99).

Synthesis of *trans*-porphodilactol (*trans*-2) and Zinc-*trans*-porphodilactol (Zn-*trans*-2)

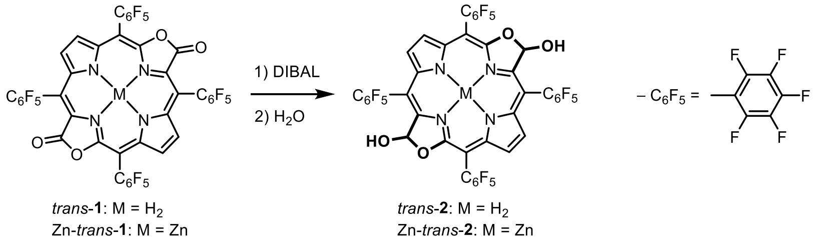

Scheme S1. Synthetic procedure of *trans*-**2** and Zn-*trans*-**2**.

Synthesis of *trans*-2. General procedure starting with *trans*-**1**.[9] Yield: 78%. ^{1}H NMR (400 MHz, $CHCl_3$-*d*): δ 8.36 (d, *J* = 4.8 Hz, 2H), 8.33 - 8.23 (m, 2H), 7.78 (d, *J* = 8.9 Hz, 2H), 3.96 (dd, J = 14.4, 10.1 Hz, 2H), -1.34 (s, 2H). M^+: Calcd for $C_{42}H_{10}F_{20}N_4O_4^+$ 1014.53443; found: 1014.03633. UV/Vis (CH_2Cl_2, 25 °C): λ_{max} (nm) (log ε): 348 (4.95), 379 (5.08), 443 (3.71), 471 (3.91), 503 (4.46), 609 (4.04), 656 (4.47), 722(5.10).

Synthesis of Zn-*trans*-2. General procedure starting with Zn-*trans*-**1**. Yield: 70%. ^{1}H NMR (400 MHz, $CHCl_3$-*d*) δ 8.12 (d, *J* = 4.4 Hz, 2H), 8.05 (d, *J* = 4.5 Hz, 2H), 7.78 (d, *J* = 9.0 Hz, 2H), 4.26 (d, *J* = 9.0 Hz, 2H). HR-MS (ESI$^+$) *m/z* $[M+H]^+$: Calcd for $C_{42}H_9F_{20}N_4O_4Zn^+$ 1078.92266; found: 1078.96253. UV/Vis (CH_2Cl_2, 25 °C): λ_{max} (nm) (log ε): 332 (5.08), 387 (5.16), 491 (3.99), 534 (4.54), 699 (4.25), 754(5.19).

Synthesis of Gd-*trans*-3.

Gd-*trans*-**3** was synthesised using a previous literature protocol with slight modification[8]. To a stirred solution of Gd-*trans*-**2** (0.025 mmol) in MeOH (10 mL), $BF_3{\cdot}Et_2O$ (0.2 mL) was added dropwise and the resulting solution was stirred at room temperature for 1 h. The volatiles were removed under vacuum, and the crude residue was purified by silica gel column chromatography using petroleum ether (PE)/ethyl acetate (EA) (v/v = 7/1) as the eluent to afford Gd-*trans*-**3** as a red powder. Yield: 88%. HR-MS (ESI$^+$) *m/z* $[M+H]^+$: Calcd for $C_{55}H_{36}CoF_{20}GdN_4O_{13}P_3^+$ 1649.98448; found: 1649.98068. UV/Vis (CH_2Cl_2, 25 °C): λ_{max} (nm) (log ε): 345 (4.99), 397 (5.15), 509 (3.99), 545 (4.50), 690 (4.11), 749(5.10).

Preparation of porphyrinoid loaded mesoporous silica nanoparticles (MSN-PS)

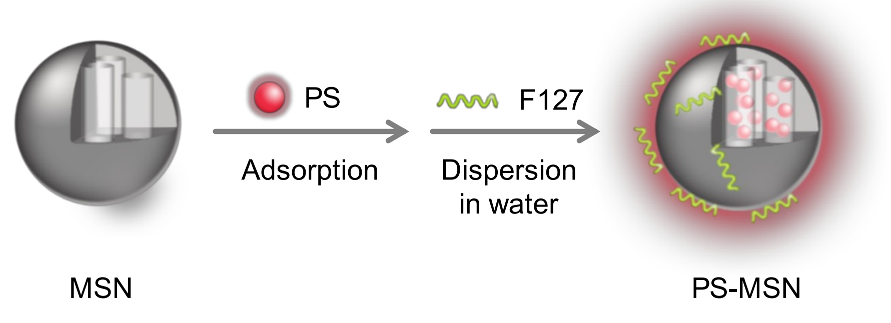

Scheme S2. Procedure for the preparation of photosensitiser (PS) loaded mesoporous silica nanoparticles (MSN-PSs).

Mesoporous silica nanoparticles (MSNs) were synthesised using a previously reported procedure.[10] In brief, MSN (25 mg) was added to a solution of THF (5 mL) containing the corresponding PS (1

μmol). The mixture was then stirred in the dark at room temperature and THF was allowed to slowly evaporate (ca. 6 h). H_2O (5 mL) was subsequently added and the resulting suspension was subjected to sonication for 20 min. The desired PS-MSNs were collected after 5 min by low-speed centrifugation (2000 $rpm \cdot min^{-1}$) and washed with water (5 mL × 2). The PS-MSNs (25 mg) were dried at 40 °C under vacuum for 24 h and dispersed using Pluronic® F-127 (100 mg) in a THF/toluene mixture (v/v = 1/1, 5 mL). The volatiles were removed under vacuum (ca. 1 h) at 35 ºC. Phosphate-buffered saline (PBS, 2 mL) was added and the suspension was stirred vigorously for 4 h at room temperature. After allowing to sit for another 24 h, the supernatant was collected and stored at 4 °C in the dark prior to use.

Characterization of the loading capacity of PS (entrapment efficiency) within the MSN-PS nanoparticles

After collecting PS-MSNs by low-speed centrifugation, the supernatant containing excessive PS was collected and extracted with DCM. The UV-vis absorption spectrum was measured and the concentration of PS was determined using the absorption maximum (A_{max}) and the corresponding extinction coefficient (ε_{max}, Table 1). The loading capacity of PS in MSN-PS was then calculated to be 84%, 79% and 80% for *trans*-**2**, Gd- *trans*-**2** and Gd-*trans*-**3**, respectively, using the following equation:

$$\text{Entrapment efficiency (\%)} = 1\text{-}\ A_{max}/(\varepsilon_{max} b n_{tot}/V)\times 100\% \qquad \text{(S1)}$$

where b stands for the optical path (b = 1 cm), and V stands for the volume of DCM; n_{tot} stands for the total amount of PS used.

Detection of reactive oxygen species (ROS)

Singlet oxygen ($^1O_2$) quantum yield (Φ_Δ) determination. The phosphorescence of PS-generated $^1O_2$ at 1270 nm was determined in air saturated $CHCl_3$ solutions. Excitation of the PS under study was achieved using either a 405 nm (for Φ_Δ determination) or 760 nm light source. 5,10,15,20-Tetrakisphenylporphyrin (H_2TPP, $\Phi_\Delta = 0.55$ in $CHCl_3$[11]) was used as reference. The Φ_Δ was calculated using the following equation:

$$\Phi_{\Delta\,S} = \Phi_{\Delta\,R}\times(I_S/I_R)\times(A_R/A_S) \qquad \text{(S2)}$$

where *I* and *A* stand for the integrated area and the absorbance at the excited wavelength (405 nm,

typically adjusted to 0.1), respectively. The subscripts S and R stand for the sample (the studied PS) and the reference (H_2TPP), respectively.

Determination of superoxide ($O_2^{\bullet-}$) production using hydroethidine (DHE) as the probe. The fluorescent probe hydroethidine (DHE) reacts with $O_2^{\bullet-}$ to afford a daughter product characterised by an intense red fluorescence emission (excitation/detected 532/610 nm). The PS-MSNs subject to analysis were suspended in water (2 mL) in the presence of DHE (10 µM). The mixed suspension was exposed to 760 nm LED light (7.5 mW·cm^{-2}), and the fluorescence at different time points (0, 20, 40, 60, 90, 120, 180 seconds) was measured with a fluorimeter. The $O_2^{\bullet-}$ quantum yield ($\Phi(O_2^{\bullet-})$) was determined using methylene blue (MB) irradiated at 635 nm, 7.5 mW·cm^{-2}, $\Phi(O_2^{\bullet-}) = 0.45$ in water[12]) as a reference per the following equation:

$$\Phi(O_2^{\bullet-})_S = \Phi(O_2^{\bullet-})_R \times (k_S/k_R) \times (A_R/A_S) \tag{S3}$$

where k is the slope of the fluorescence emission intensity at 610 nm over time, and A is the absorbance at the irradiation wavelength (adjusted to be ca. 0.25). The subscripts S and R stand for the sample (PS) and the reference (MB), respectively.

Determination of hydroxyl radical (HO•) generation by EPR. 5-*Tert*-butoxycarbonyl-5-methyl-1-pyrroline-N-oxide (BMPO) was used as a radical trap since it forms distinct adducts with HO• (BMPO-OH)[13]. The PS-MSNs under study were suspended in H_2O (500 µL, at a PS concentration of $A_{760mn} = 0.25$) in the presence of BMPO (25 mM). Each suspension was photo-irradiated for 10 min (760 nm, 7.5 mW·cm^{-2}). The EPR signal of each sample was then recorded using an EPR spectrometer at room temperature.

Measurement of the photothermal effect

Estimating photothermal conversion efficiency. To evaluate the photothermal efficacy of the MSN-PSs under study, they were suspended in H_2O (200 µL) at a PS concentration of 100 µg·mL^{-1} and photo-irradiated using a 760 nm LED (100 mW·cm^{-2}) for 6 min. The photothermal effect was analysed in real-time using a thermometer at different time points. The photothermal conversion coefficient (η) was calculated according to the following equation:

$$\eta = \frac{hA\Delta T_{max}}{I} = \frac{hA\Delta T_{max} - Q_s}{I(1-10^{-A_\lambda})} \tag{S4}$$

where $\Delta T_{\max}$ = temperature difference between maximum stable temperature and the initial

temperature; I is the power of light used to irradiate the sample; Q_S = heat induced by the light absorption of water (solvent), A_λ is the absorbance of the PS suspension at 760 nm, h is the heat transfer coefficient and A is the surface area of the system. The value of hA could then be derived using equation S5:

$$t = \frac{\sum_i m_i C_{p,i}}{-hA} \ln\theta \approx \frac{m_w C_{p,w}}{-hA} \ln\theta \quad \text{(S5)}$$

where lnθ = ratio of ΔT to ΔT_{max}; m - weight (g). C_p = specific heat of the component in the suspension, which can be approximated to the values for water (m_w and $C_{p,w}$ in the equation). The term hA was derived from the slope of a plot of cooling time t vs lnθ.

IR thermal imaging. IR thermal images were obtained with an IR thermographic camera (FLIR E40) using the experimental conditions detailed above.

Cell culture studies

4T1 cells and HeLa cells were used and cultured in Dulbecco's modified Eagle medium (DMEM, Corning) supplemented with 10% fetal bovine serum (FBS), 1% penicillin and streptomycin. Cells were grown at 37 °C in a humidified atmosphere containing 5% CO_2.

***In vitro* cell imaging**

HeLa cells were used for cellular imaging experiment due to their appropriate size. Cells were seeded on sterile glass coverslips in cell culture dishes containing complete media and allowed to grow to ~ 80% confluence. The PS-MSNs (4 μM·mL^{-1} PS dissolved in PBS) under study were then added. After 12 h, LysoTracker Green® DND-26 (75 nM) was added and the cells incubated for another 30 min. The cells were washed with PBS (3 times) before imaging and the NIR fluorescence signal of the PS was collected through a Samrock 776 nm long-pass filter, with excitation effected at 740 nm using a YSL supercontinuum laser source. The fluorescence corresponding to the LysoTracker Green® probe was collected using a Semrock 525/50 nm bandpass filter with excitation effected at 488 nm using a laser.

***In vitro* cytotoxicity studies**

Dark cytotoxicity. 4T1 cells were seeded in flat-bottomed 96-well plates (10^4 cells per well) with DMEM media (200 μL) for 24 h. Cells were incubated with the PS-MSN under study (0 - 16 μM of the PS) for 24 h, then washed with PBS (3 times) and cultured for another 24 h. The cells were then washed with PBS (3 times). To each well, 10 μL of a Cell Counting Kit-8 (CCK-8) solution and 90 μL

of PBS were added. After 2 h, the absorbance at 450 nm was read using a 96-well plate reader. The viability of 4T1 cells was calculated using the following equation:

$$CV = (A_s - A_b)/(A_c - A_b) \times 100\% \quad \text{(S6)}$$

where CV = cell viability, A_s, A_c and A_b represent the absorbance of cells incubated with PS, cell control (no PS), and blank control (wells containing neither cells nor the studied PS), respectively.

Determination of the photocytotoxicity of each PS (normoxic conditions). 4T1 cells were seeded and cultured with PS-MSN according to the above dark cytotoxicity protocol. After incubating for 24 h with PS-MSN (0-16 μM in PS), the cells were washed with PBS (3 times) and irradiated for 30 min in PBS (100 μL) using a 760 nm LED (7.5 mW·cm^{-2}). The PBS was then replaced with fresh culture media (200 μL) and the cells were cultured in the dark for another 24 h. Cell viability was determined using the CCK-8 assay per equation S6.

Determination of the photocytotoxicity of each PS under hypoxic conditions. 4T1 cells were seeded and cultured with the chosen PS and the dark cytotoxicity protocol outlined above was followed. After being incubated with the PS-MSN under study (0-16 μM in the PS) for 24 h, the cells were washed with PBS (3 times) and sealed in an AnaeroPouch-Bag (MGC AnaeroPouch-Bag C-11) for 6 h to create an anoxic environment. Cells were then irradiated for 30 min (760 nm LED, 7.5 mW·cm^{-2}) and then cultured in fresh culture media (200 μL) under normoxic conditions for another 24 h. Cell viability was determined using the CCK-8 assay per equation S6 above.

Photocytotoxicity experiments using ROS scavengers. Sodium azide (NaN_3), Mn(II)-tetrakis(3-N-methylpyridiniumy)porphyrin (MnTPyP), dimethyl sulfoxide (DMSO) and sodium pyruvate (NaP) were used as ROS scavengers for $^1O_2$, $O_2^{\bullet -}$, OH• and H_2O_2, respectively. 4T1 cells were seeded and cultured with the MSN-Gd-*trans*-**2** using the same conditions as in the dark cytotoxicity experiments. After 24 h incubation with MSN-Gd-*trans*-**2** (8 μM of Gd-*trans*-**2**), the cells were incubated for another 2 h with the corresponding ROS scavengers (NaN_3: 10 mM, MnTPyP: 100 μM, NaP: 10 mM, and DMSO: 0.28 M). The cells were then washed with PBS (3 times) and irradiated for 30 min in PBS (100 μL) with a 760 nm LED (7.5 mW·cm^{-2}). The PBS was then replaced with fresh culture media (200 μL) and the cells were cultured in the dark for another 24 h. Cell viability was determined using the CCK-8 assay per equation S6 above.

Determining ROS generation levels in vitro

In vitro ROS detection. Total in vitro ROS generation in live cells was determined using 2',7'-dichlorodihydrofluorescein diacetate (H2DCFDA), an indicator that reacts with cellular ROS to provide an increase in the fluorescence emission intensity (excitation/emission maxima 504/529 nm). 4T1 cells were seeded on sterile glass coverslips in cell culture dishes containing complete media. The cells were then cultured for 12 h. The PS-MSNs under study (4 μM·mL^{-1} PS dissolved in PBS) was then added. After 12 h, H2DCFDA (10 μM) was added and the cells incubated for another 30 min. The cells were washed with PBS (3 times) and exposed to light (760 nm, 7.5 mW·cm^{-2}) for 30 min. Confocal fluorescence microscopy was used to visualize ROS generation in vitro by monitoring the increase in fluorescence within the cells. Each sample was excited at 488 nm and detected through a 525/50 bandpass filter. This will be referred to as Procedure 1.

Detection of $^1O_2$ in vitro. Singlet oxygen ($^1O_2$) generation was detected using Singlet Oxygen Sensor Green (SOSG), a probe that reacts with $^1O_2$ to produce an increase in fluorescence emission intensity (excitation/emission maxima 504/525 nm). The full experimental protocol followed Procedure 1.

Detection of $O_2^{\bullet-}$ in vitro. Superoxide ($O_2^{\bullet-}$) generation was detected using DHE. The full experimental protocol followed Procedure 1. For DHE fluorescence, the samples under study were excited at 543 nm and detected through a 595/50 bandpass filter.

Apoptosis analyses

Western blot analysis. Western blot protein expression of poly ADP-ribose polymerase (PARP) and Caspase 3 of 4T1 cells was performed and quantified as follows: 4T1 cells were seeded and cultured in a 6-well plate for 12 h, and incubated with Gd-*trans*-**2** (4 μM) or PBS (control) for 24 h. After 3 washing steps (PBS), cells were subjected to photo-irradiation (760 nm, 7.5 mW·cm^{-2}) for 30 min, except for the dark control group (Gd-*trans*-**2** (4 μM) only). After incubating for an additional 24 h, the cells were washed with PBS (3 times), followed by centrifugation at 1200 rpm for 5 min. Proteins were extracted from the cells using a commercially available total protein extraction kit for standard western blotting. Target proteins were detected with primary antibodies recognizing PARP or Caspase 3, respectively. Images were acquired using a Bio-Rad ChemiDoc Touch imaging system and quantified using the Image J software.

Flow cytometry for apoptosis. 4T1 cells were seeded and cultured in 6-well plates for 12 h. After incubation with Gd-*trans*-**2**-MSN (8 μM in Gd-*trans*-**2**) for 24 h, cells were washed with PBS (3 times) followed by photo-irradiation (760 nm, 7.5 mW·cm^{-2}) for 30 min. After incubating for an additional

24 h, the cells were stained with the Annexin V-FITC/PI apoptosis kit (Solarbio®) and quantified by flow cytometry carrying out 3 different runs to allow for viable statistical analyses.

***In vivo* imaging experiments**

***In vivo* NIR fluorescence imaging.** Five-week-old male BALB/c mice were obtained from Peking University First Hospital (Beijing, China) and were housed under standard environmental conditions for in vivo studies. **All animal procedures were approved by the Institutional Animal Care and Use Committee of Peking University.** Mice were randomly selected from their cages for each experiment. A syngeneic model was established by subcutaneously inoculating 1×10^6 4T1 cells (200 μL PBS) into the right flank of each BALB/c mouse. 4T1-tumor-bearing mice were intravenously injected (tail vein) with PBS (250 μL) or PS-MSN (1 $mg{\cdot}kg^{-1}$ of the PS-MSN in 250 μL PBS). In vivo fluorescence imaging was performed at different time points (0, 6, 24, and 48 h) to determine the IVIS spectrum (excitation/emission wavelength = 740/780 nm). The mice were then sacrificed 48 h post-injection to acquire the ex vivo fluorescence images used to determine the PS-MSN biodistributions.

***In vivo* photoacoustic imaging (PAI).** 4T1 tumor-bearing mice were randomly selected and intravenously injected (tail vein) with PBS (250 μL) or PS-MSN (1 $mg{\cdot}kg^{-1}$ of the PS). In vivo PA imaging was performed with optoacoustic tomography imaging platform at 0 and 24 h post-injection.

***In vivo* phototoxicity experiments**

***In vivo* photothermal effect.** 4T1 tumor-bearing mice were randomly selected and injected (i.v.) with PBS (250 μL) or MSN-PS (1 $mg{\cdot}kg^{-1}$ of the PS). At 24 h post-injection, the tumors were irradiated with 760 nm light (100 $mW{\cdot}cm^{-2}$) for 5 min. The temperature change and IR thermographic map were then determined using an infrared thermal imaging camera.

Tumor-inhibition efficacy. 4T1 tumor-bearing mice were randomly divided into 6 groups: (1) PBS + dark, (2) Gd-*trans*-**2** + dark, (3) PBS + light, (4) *trans*-**2** + light, (5) Gd-*trans*-**2** + light, and (6) Gd-*trans*-**3** + light. Each group consisted of 7 mice. The mice were intravenously injected (tail vein) with the indicated solution or corresponding MSN-PS suspension (1 $mg{\cdot}kg^{-1}$ in the PS in 250 μL PBS) on Day 0. 24 h post-injection, the tumors of the mice in the light groups (groups (3)-(6)) were photo-irradiated with 760 nm light (100 $mW{\cdot}cm^{-2}$) for 5 min. The tumor sizes were measured with a digital caliper twice a week and the tumor volume (*V*) was calculated using the following equation:

$$V = (\text{length} \times \text{width}^2)/2 \quad \text{(S7)}$$

The body weight of each mouse was monitored twice a week. Mice were sacrificed on day 28 and the tumors and major organs were sectioned for hematoxylin-eosin (H&E) staining and immunohistochemistry analysis.

Statistical analyses

The data was expressed as the mean ± SD. The experimental data was subjected to a one-way ANOVA statistical analysis. The data were classified with p values and denoted by * for $p < 0.05$ and ** for $p < 0.01$.

2. Additional Figures, Spectra and Tables

Table S1 Calculated $\Delta E_{S\text{-}T}$, HLgap, and NICS values, and N-H ^{1}H NMR signals

	F_{20}TPP	F_{20}TPPlac	*trans*-**1**	*trans*-**2**
ΔE_{ST} /eV	0.73	1.13	0.84	1.50
HOMO /eV	-3.33	-3.69	-4.09	-3.69
LUMO /eV	-6.18	-6.35	-6.42	-5.70
HLgap /eV	2.86	2.65	2.34	2.01
NICS(1) /ppm	-13.45	-12.07	-12.16	-11.46
NICS(0) /ppm	-14.45	-12.90	-13.03	-12.26
N-H ^{1}H NMR /ppm	-2.91 [a]	-1.94 [b]	-2.12 [c]	-1.35

[a] From ref. 13[14]. [b] From ref. 14[15]. [c] From ref. 15[16].

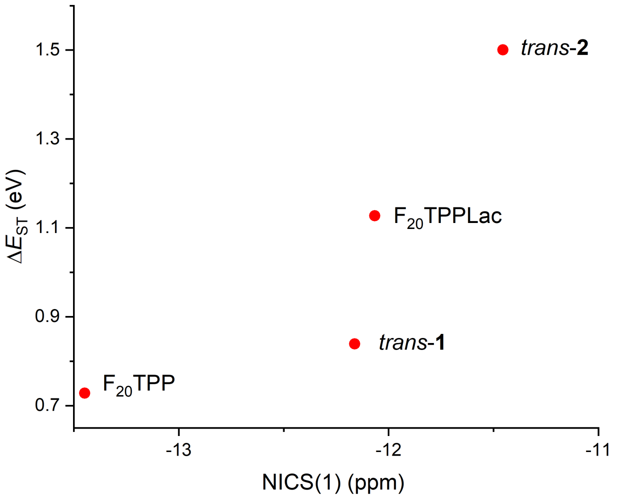

Fig. S1 Relationship between calculated $\Delta E_{S\text{-}T}$ spectra and calculated NICS(1) values for porphyrin (F_{20}TPP), porpholactone (F_{20}TPPLac), *trans*-porphodilactone (*trans*-**1**) and *trans*-porphodilactol (*trans*-**2**).

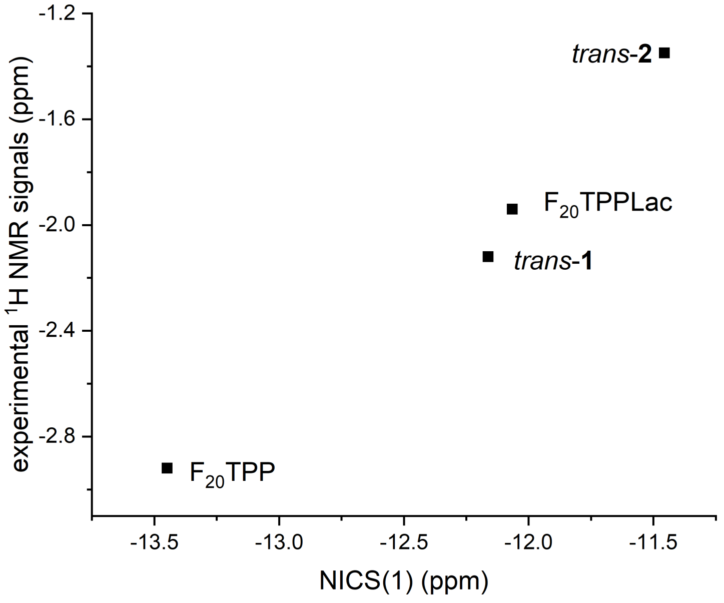

Fig. S2 Relationship between the chemical shifts of the inner N–H proton signals observed in the ^{1}H NMR spectra and the calculated NICS(1) values of porphyrin (F_{20}TPP), porpholactone (F_{20}TPPLac), *trans*-porphodilactone (*trans*-**1**) and *trans*-porphodilactol (*trans*-**2**).

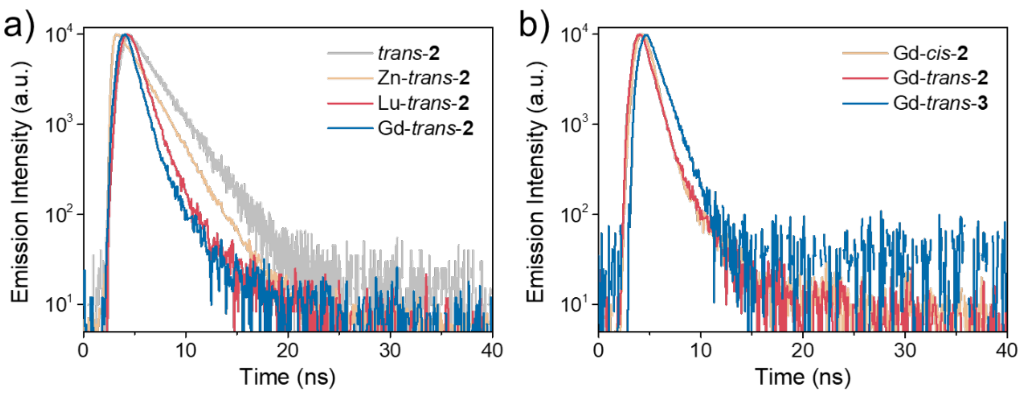

Fig. S3 Fluorescence decay curves of (a) the *trans*-**2** series of porphyrinoids (*trans*-**2**, Zn-*trans*-**2**, Lu-*trans*-**2** and Gd-*trans*-**2**), and (b) the set of Gd(III) complexes considered in the present study (Gd-*cis*-**2**, Gd-*trans*-**2** and Gd-*trans*-**3**) in DCM at room temperature (λ_{ex} = 405 nm, λ_{em} = the emission maximum).

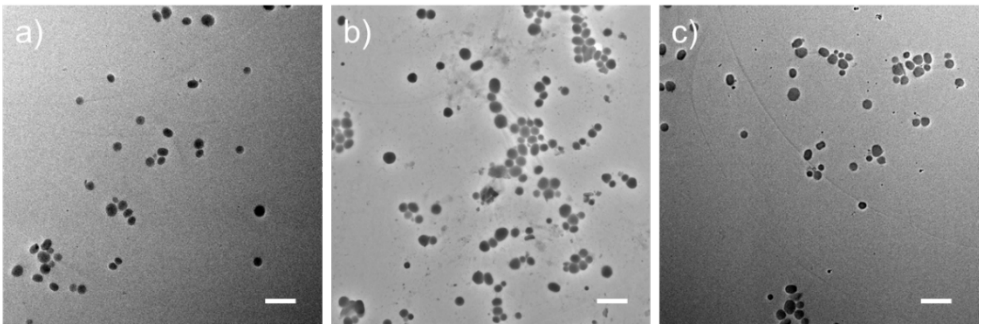

Fig. S4 Transmission electron microscopy (TEM) images at 25k× magnification of (a) MSN-*trans*-**2**, (b) MSN-Gd-*trans*-**2**-MSN and (c) Gd-*trans*-**3**-MSN. Scale bar: 1 μm.

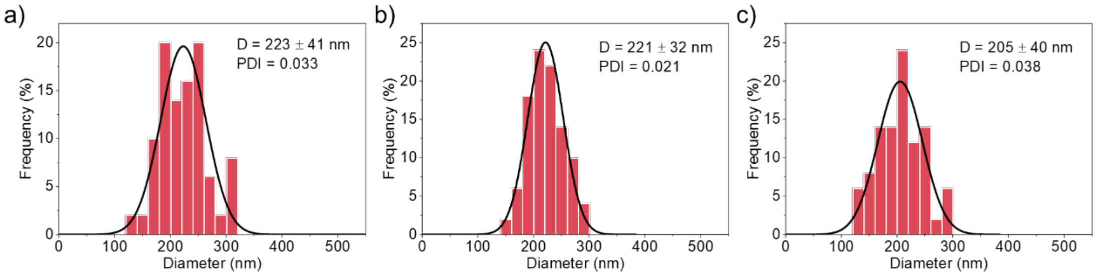

Fig. S5 Size distribution of (a) MSN-*trans*-**2**, (b) MSN-Gd-*trans*-**2**-MSN and (c) Gd-*trans*-**3**-MSN as inferred from TEM images. 50 particles of each construct were randomly selected and the diameter (D) was measured using ImageJ. The polydispersity index (PDI) was calculated as PDI = σ^2/D_a^2 (σ and D_a stand for the standard deviation and the average value of particle diameter, respectively)

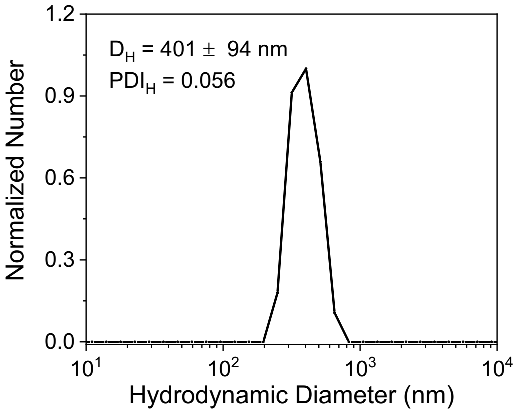

Fig. S6 Dynamic light scattering (DLS) of MSN-Gd-*trans*-**2**. D_H stands for hydrodynamic diameter and PDI was calculated as PDI = σ^2/D_H^2.

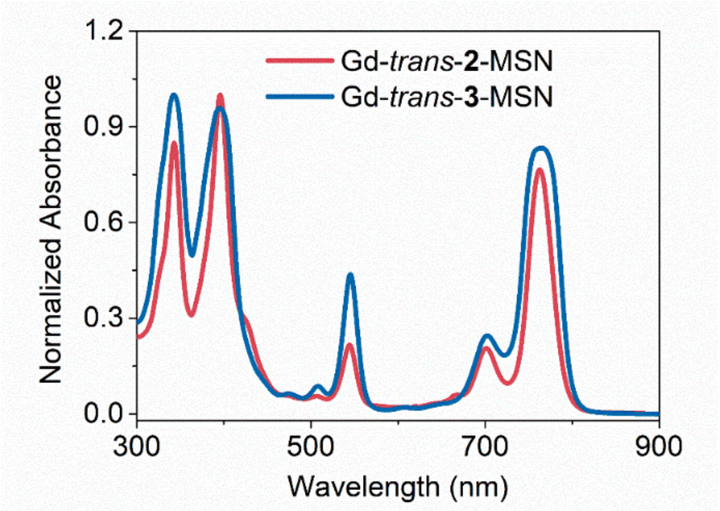

Fig. S7 Normalised UV-Vis absorption spectra of Gd-*trans*-**2**-MSN and Gd-*trans*-**3**-MSN recorded in H_2O at room temperature.

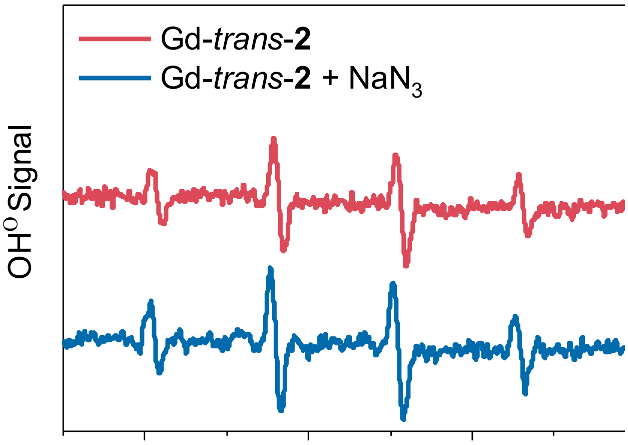

Fig. S8 OH• generation from MSN-Gd-*trans*-**2** (500 μL, at a PS concentration of A_{760nm} = 0.25) promoted by light irradiation in the absence and presence of NaN_3 (10 mM) as determined by EPR analysis using BMPO (25 mM) as the spin-trap. Irradiation condition: 760 nm,7.5 mW·cm^{-2}, 10 min.

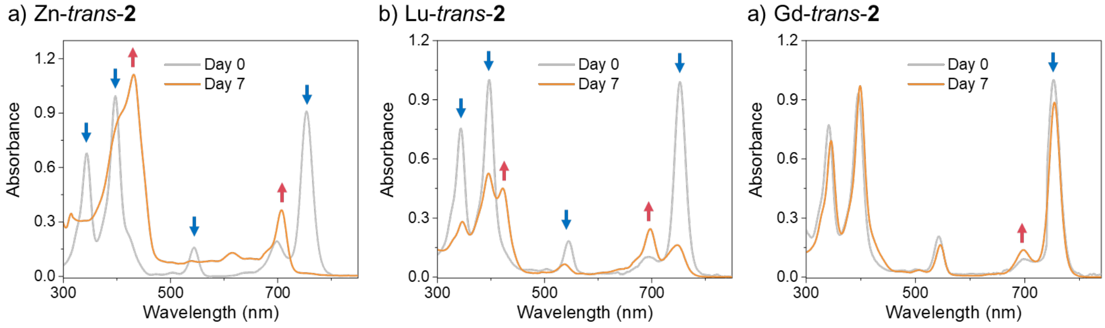

Fig. S9 UV-Vis absorption spetctra of metal porphodilactols in DCM recorded on Day 0 and Day 7. (a) Zn-*trans*-**2**, (b) Lu-*trans*-**2**, and Gd-*trans*-**2**. Solutions were stored at room temperature in the dark with no precautions taken to exclude air.

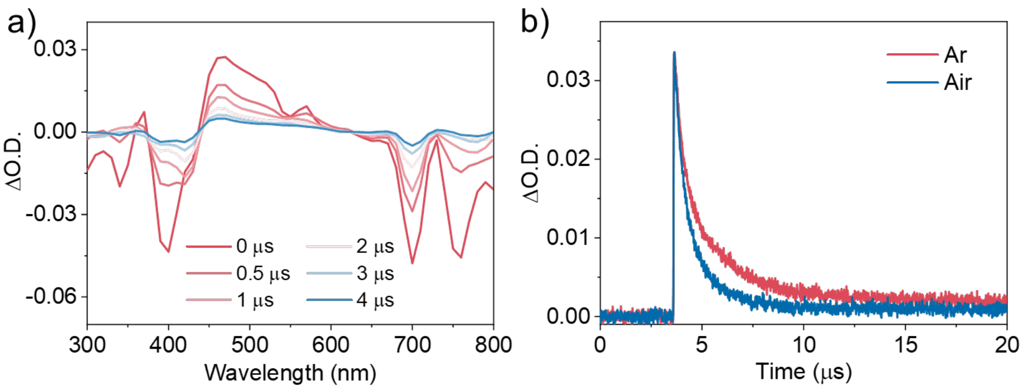

Fig. S10 a) Transient absorption difference spectra of Gd-*trans*-**3** measured at several delay times in deaerated toluene (A_{355nm} = 0.25 λ_{ex} = 355 nm). b) Decay traces for Gd-*trans*-**3** in deaerated (red) and air-saturated (blue) toluene at 470 nm. The intensity of the signal under the ambient atmosphere conditions is normalized to that in deaerated toluene.

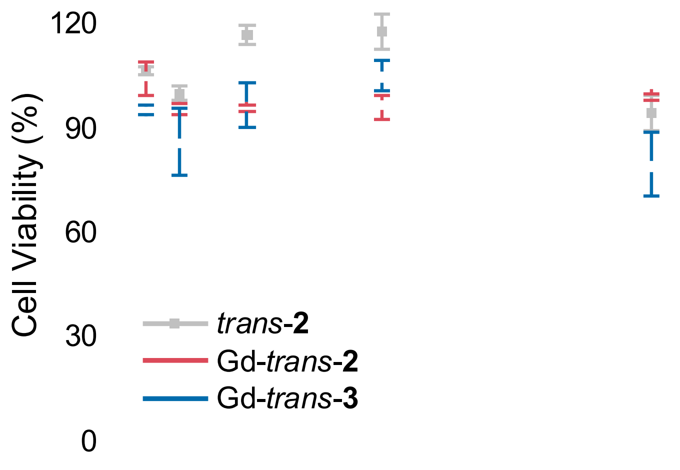

Fig. S11 Evaluation of the dark cytotoxicity of MSN-*trans*-**2** (gray), MSN-Gd-*trans*-**2** (red) and MSN-Gd-*trans*-**3** (blue) in 4T1 cells. determined using the CCK-8 assay.

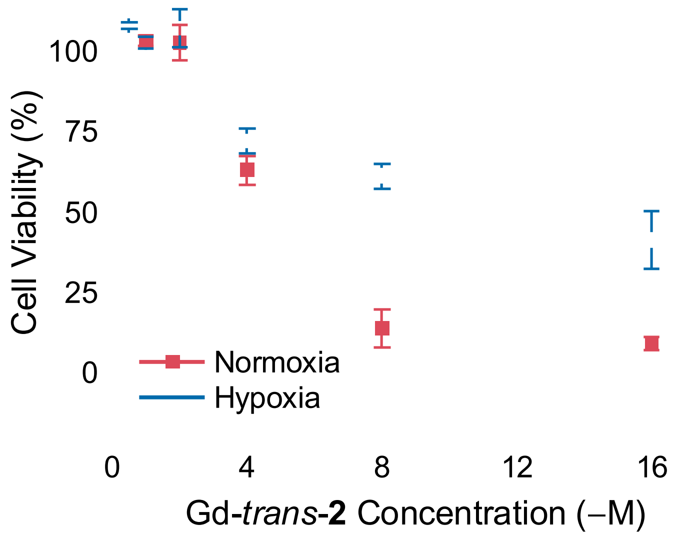

Fig. S12 Photocytotoxicity of MSN-Gd-*trans*-**2** in 4T1 cells under normoxic (red) and hypoxic (blue) conditions with photo-irradiation at 760 nm (7.5 mW·cm^{-2}, 30 min). Determined using the CCK-8 assay.

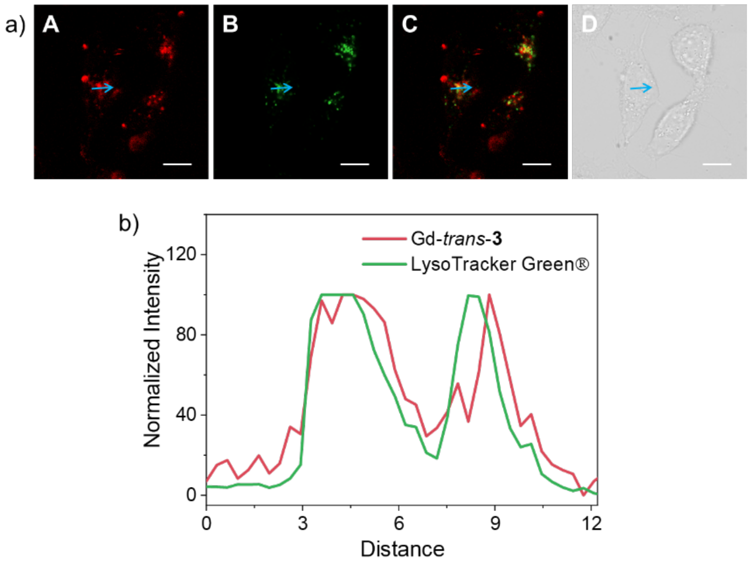

Fig. S13 a) Fluorescence colocalisation images of HeLa cells incubated with MSN-Gd-*trans*-**3** (5 μM Gd-*trans*-**3**) and LysoTracker® Green (75 nM). Columns A-D: MSN-Gd-*trans*-**3** (λ_{ex} = 740 nm, λ_{em} = 776 nm long-pass), LysoTracker® Green (λ_{ex} = 488 nm, λ_{em} = 525/50 nm bandpass), merged image of A and B, and differential interference contrast (DIC) image. Scale bar = 25 μm. b) The corresponding fluorescence profile analysis.

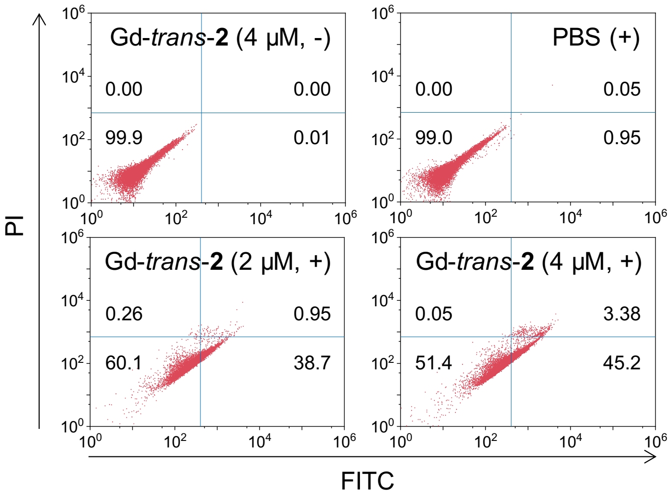

Fig. S14 Flow cytometry analysis of 4T1 cells treated with Gd-*trans*-**2**-MSN (4 μM, -), PBS (+),Gd-*trans*-**2**-MSN (2 μM, +) or Gd-*trans*-**2**-MSN (4 μM, +) using an Annexin V-FITC/PI apoptosis assay. The quadrant from the lower left to the upper left (counterclockwise) represent healthy, early apoptotic, late apoptotic, and necrotic cells, respectively. The percentage of cells in each quadrant is shown on the graphs. Here, + and - indicate experiments carried out in the presence and absence of photo-irradiation, respectively.

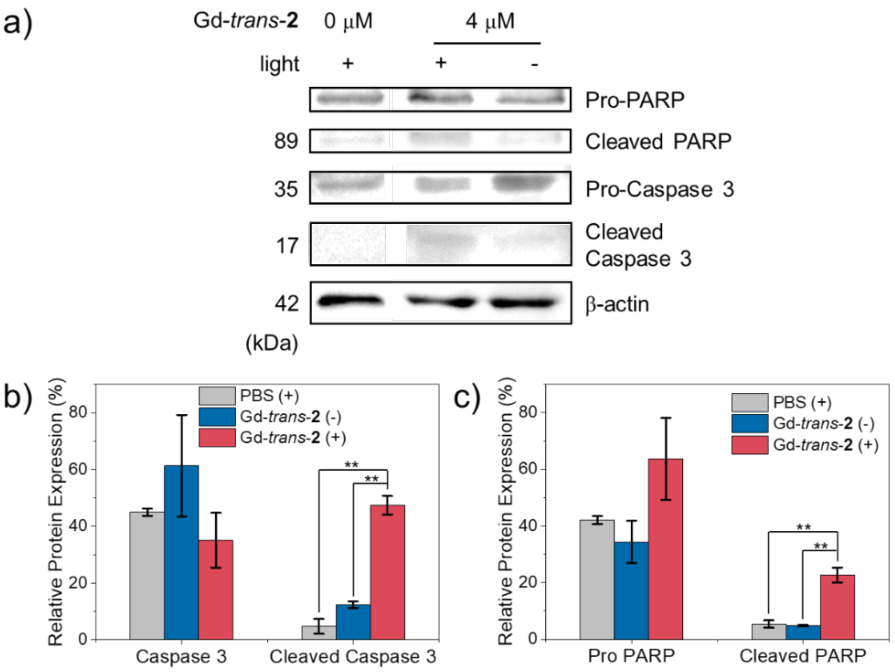

Fig. S15 a) Western blot analysis for PARP and Caspase 3 following treatment with PBS (+), MSN-Gd-*trans*-**2**-MSN (4 μM, -), and MSN-Gd-*trans*-**2**-MSN (4 μM, +), respectively. Here, + and - indicate experiments carried out in the presence and absence of photo-irradiation, respectively. Quantification analysis of (b) Caspase 3 and (c) PARP expression. ** $p < 0.01$.

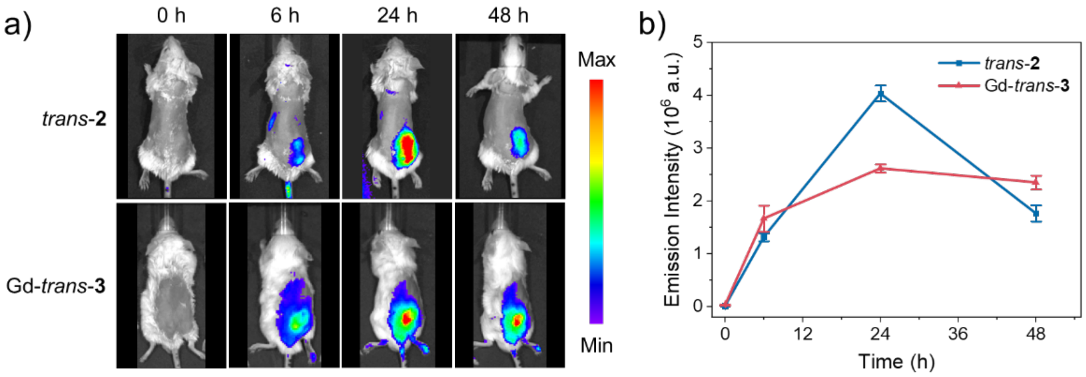

Fig. S16 (a) *In vivo* NIR fluorescence imaging and (b) emission intensity changes at the tumour site of 4T1-tumour-bearing mice after treatment with MSN-*trans*-**2**, and MSN-Gd-*trans*-**3** *via* intravenous injection (1 mg/kg of PS, tail vein; λ_{ex} = 740 nm, λ_{em} = 780 nm).

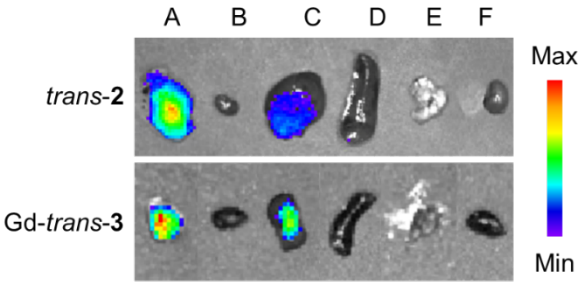

Fig. S17 *Ex vivo* fluorescence images recorded 24 h after injection with MSN-*trans*-**2** and MSN-Gd-*trans*-**3** (1 mg/kg of PS). A-F: tumor, heart, liver, spleen, lung, kidney.

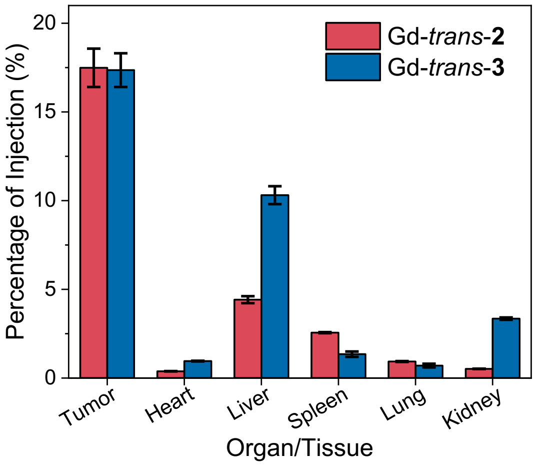

Fig. S18 Distribution of Gd^{3+} complexes in the tumour and main organs (heart, liver, spleen, lung and kidney) determined by ICP-MS. Mice were dissected 24 h after drug injection.

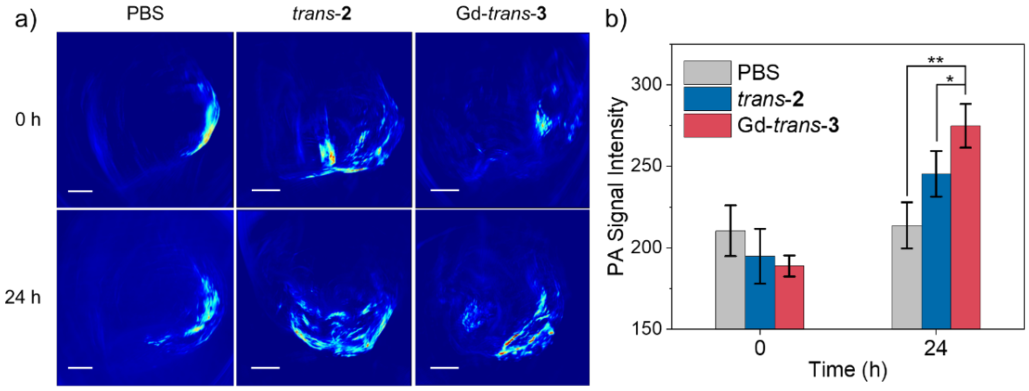

Fig. S19 a) *In vivo* PA imaging of 4T1-tumour-bearing mice before (0 h) and 24 h post-injection with the indicated MSN-PS. b) Signal intensity changes in the tumour region (n = 3, mean ± SD, * p < 0.05, ** p < 0.01). Tail vein injection; 1 mg/kg PS; λ_{ex} = 760 nm.

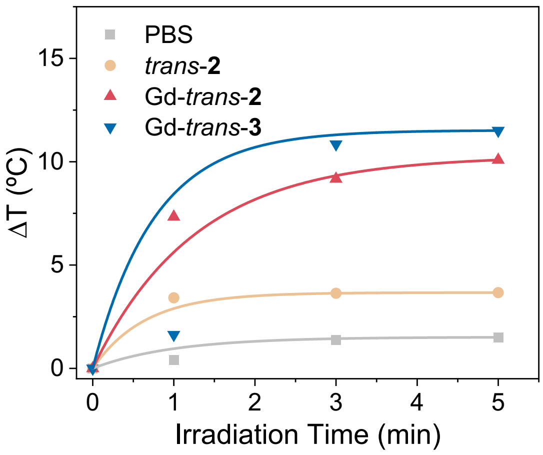

Fig. S20 Temperature changes on the tumour surface of 4T1-tumour-bearing mice that have been subjected to laser irradiation at different time points (0, 1, 3, 5 min) at 24 h post-injection of PBS, MSN-*trans*-**2**, MSN-Gd-*trans*-**2** or MSN-Gd-*trans*-**3** (1 mg/kg; tail vein injection; λ_{ex} = 760 nm, 100 mW·cm^{-2}). Measured from the IR thermal images shown in Fig. 7a of the main text.

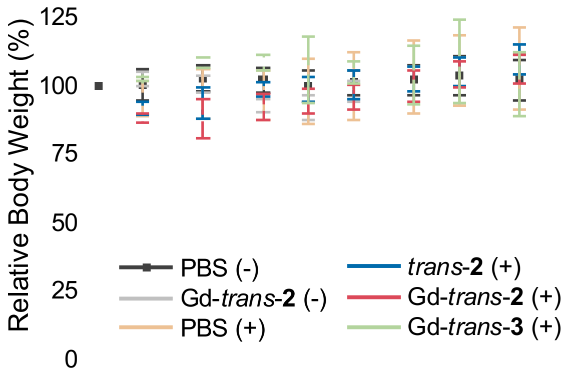

Fig. S21 Relative body weights of mice in different groups during treatment (n = 7, mean ± SD). Blue and red arrow represents the injection (0) and irradiation (1st) days, respectively; + and - denote with and without photo-irradiation (λ_{ex} = 760 nm, 100 mW·cm^{-2}, 5 min), respectively.

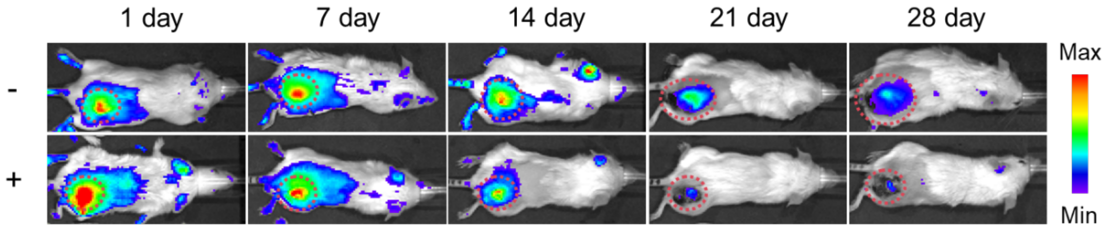

Fig. S22 *In vivo* NIR fluorescence images recorded on days 1, 7, 14, 21, and 28 day post-injection of Gd-*trans*-**2**. Animals were injected intravenously once on day 0, 1 mg/kg Gd-*trans*-**2**, tail vein; λ_{ex} = 740 nm, λ_{em} = 780 nm. "+" and "-" denote with and without photo-irradiation (λ_{ex} = 760 nm, 100 mW·cm^{-2}, 5 min), respectively.

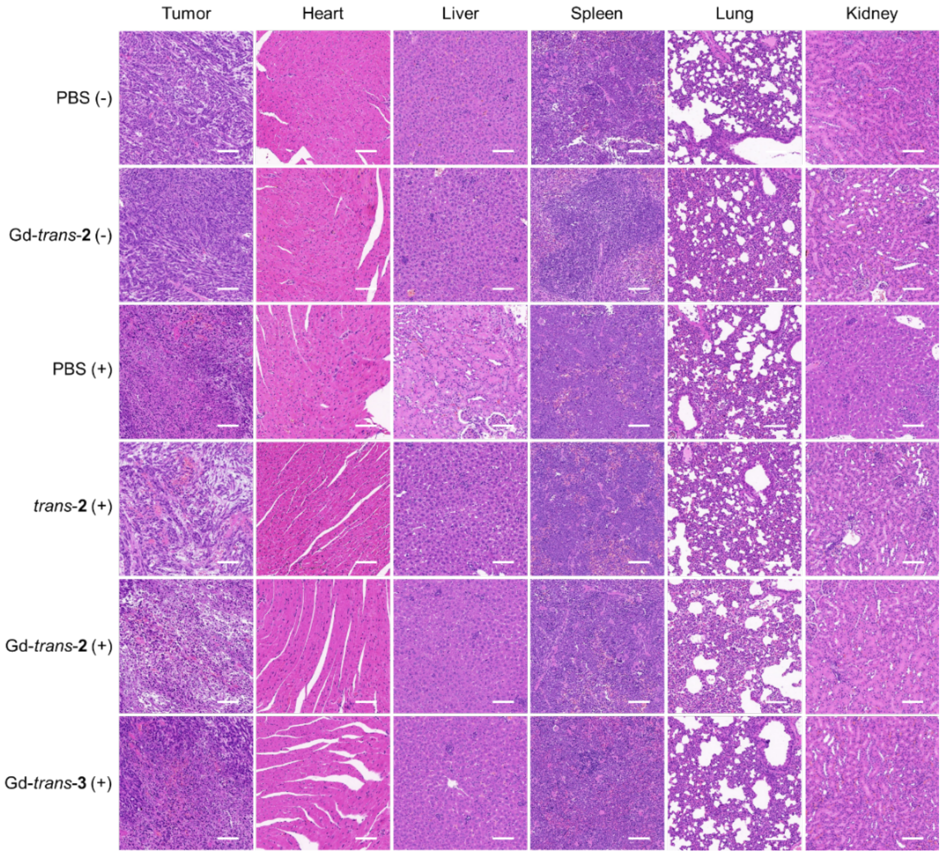

Fig. S23 H&E staining images of tumour and primary organs after treatment with the indicated agents of blank (PBS). Here + and - refer to experiments carried out with and without photo-irradiation, respectively. The scale bar is 100 μm.

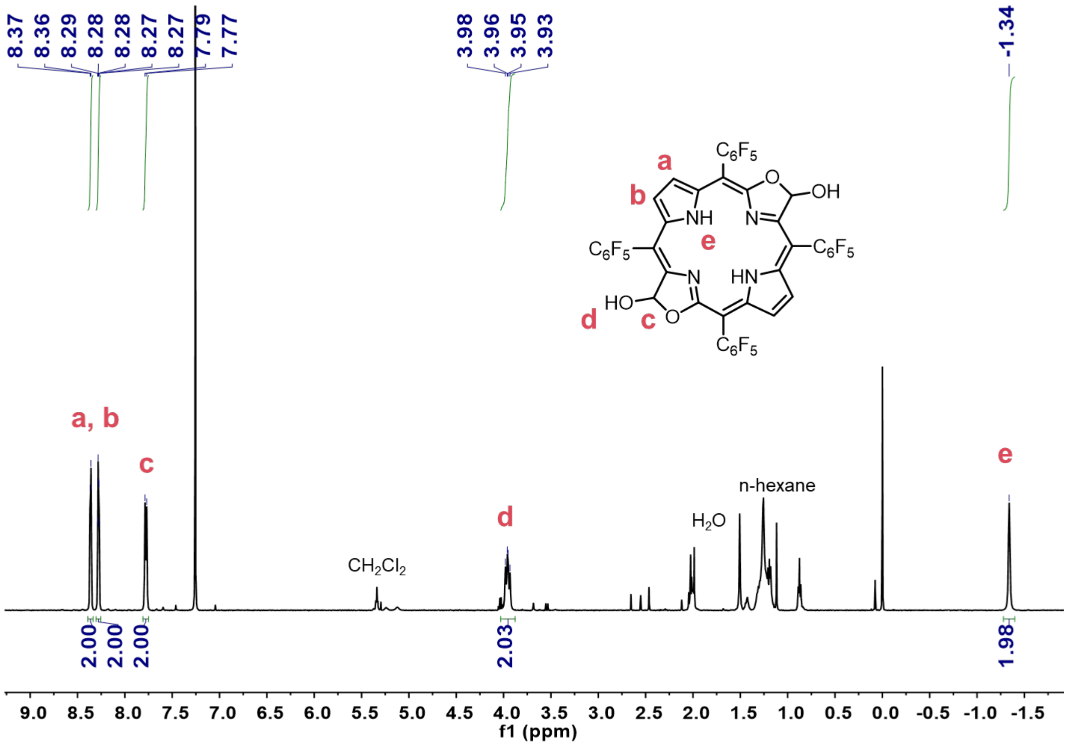

Fig. S24 ^{1}H NMR spectrum of *trans*-**2** ($CDCl_3$, 400 MHz).

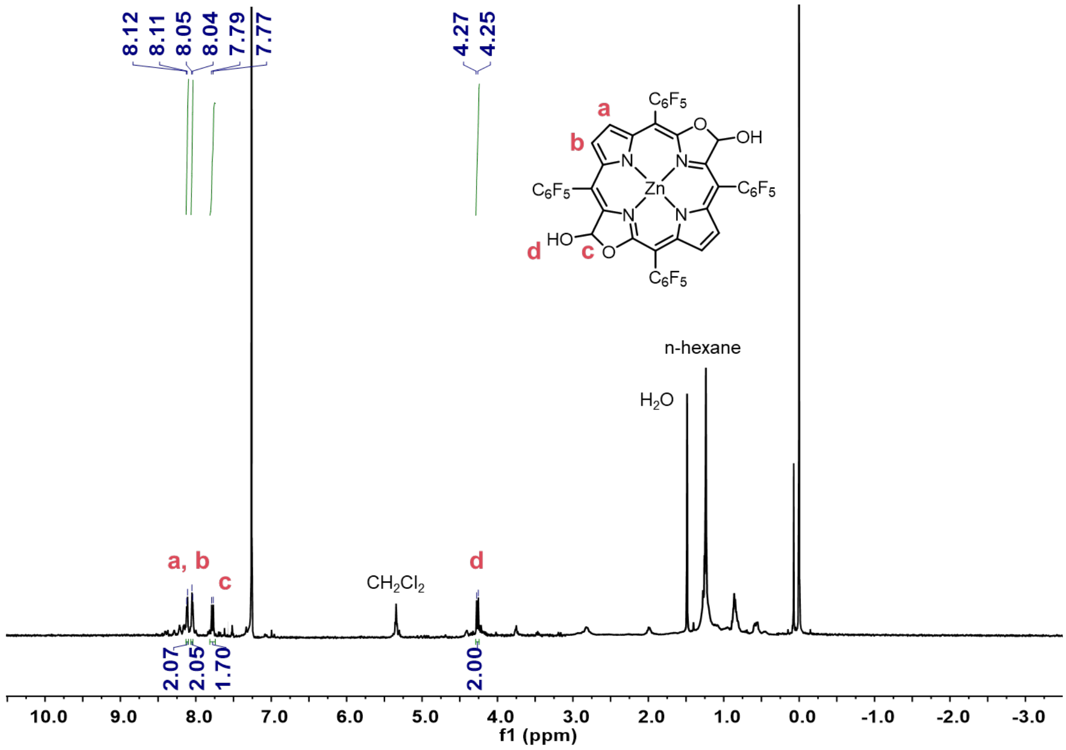

Fig. S25 ^{1}H NMR spectrum of Zn-*trans*-**2** ($CDCl_3$, 400 MHz).

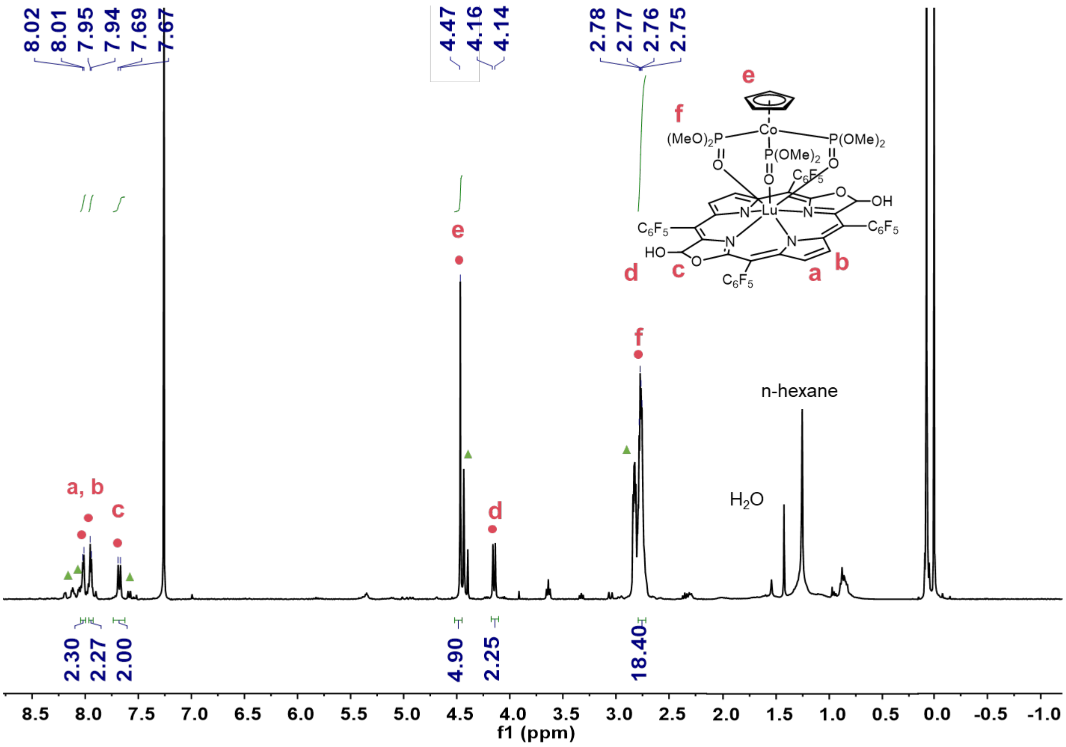

Fig. S26 ^{1}H NMR spectrum of Lu-*trans*-**2** ($CDCl_3$, 400 MHz). The two groups (highlighte with red circles and green triangles) of proton signals are ascribed to stereoisomers (β-hydroxyl groups above or below the plane of the macrocyclic ring) in accord with our previous report.[17]

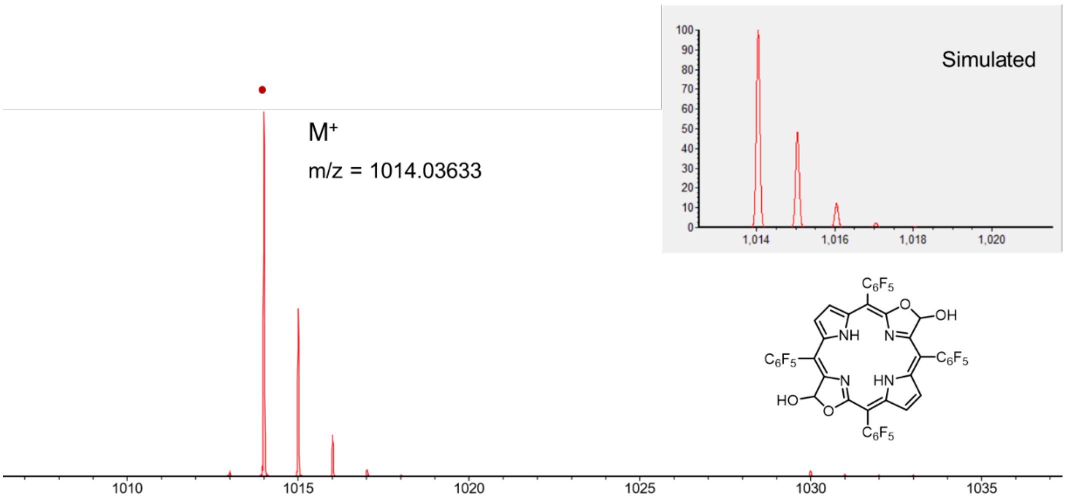

Fig. S27 HR-MS spectrum (ESI$^+$) of *trans*-**2**. Inset shows the simulated spectrum.

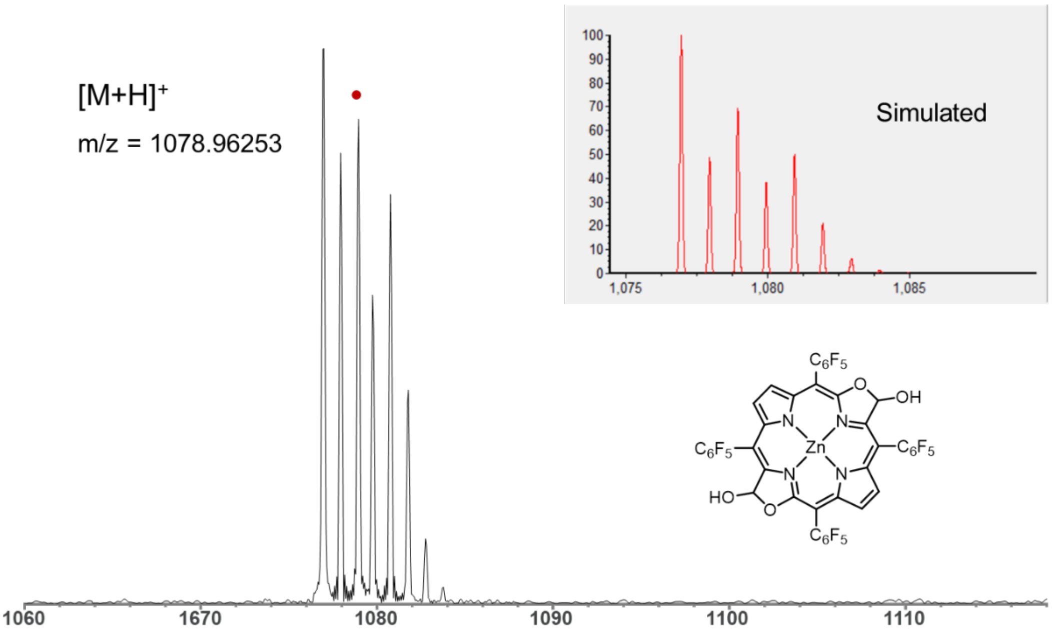

Fig. S28 HR-MS spectrum (ESI$^+$) of Zn-*trans*-**2**. Inset shows the simulated spectrum.

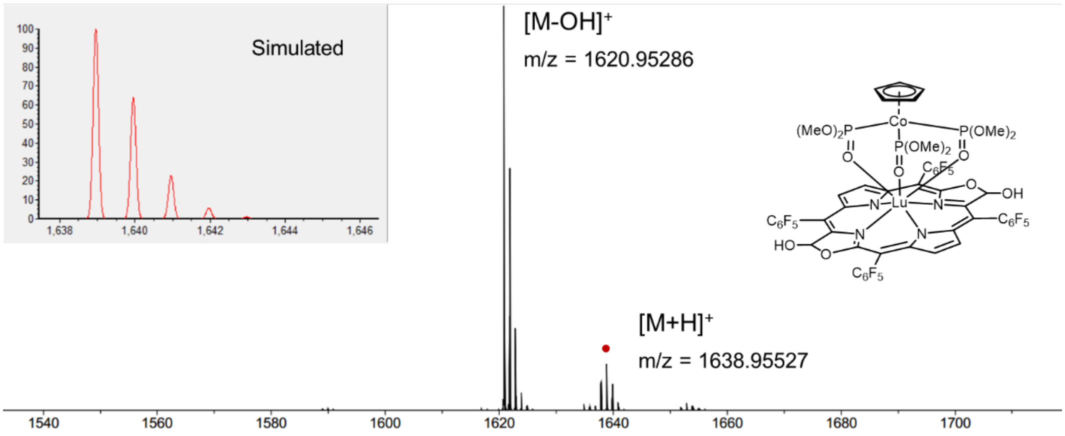

Fig. S29 HR-MS spectrum (ESI$^+$) of Lu-*trans*-**2**. Inset shows the simulated spectrum.

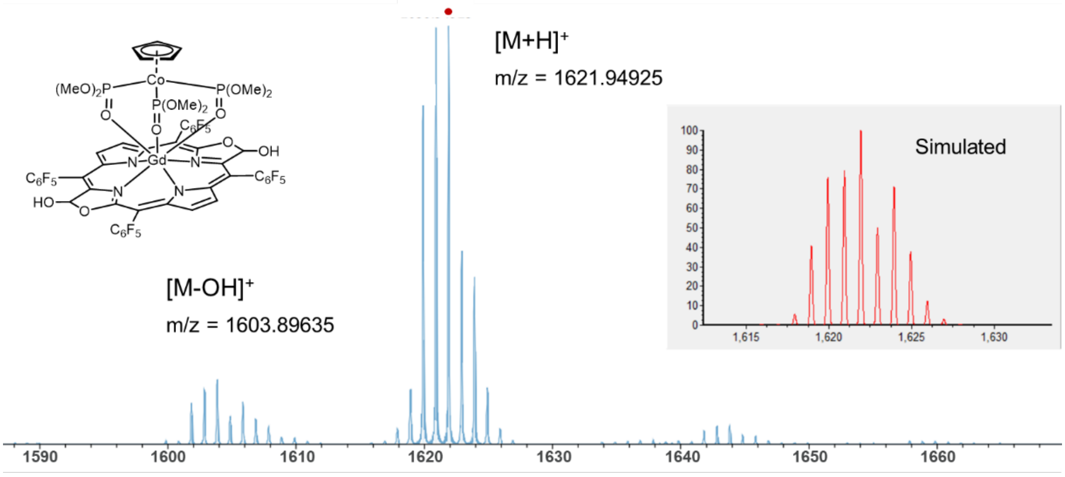

Fig. S30 HR-MS spectrum (ESI+) of Gd-*trans*-**2**. Inset shows the simulated spectrum

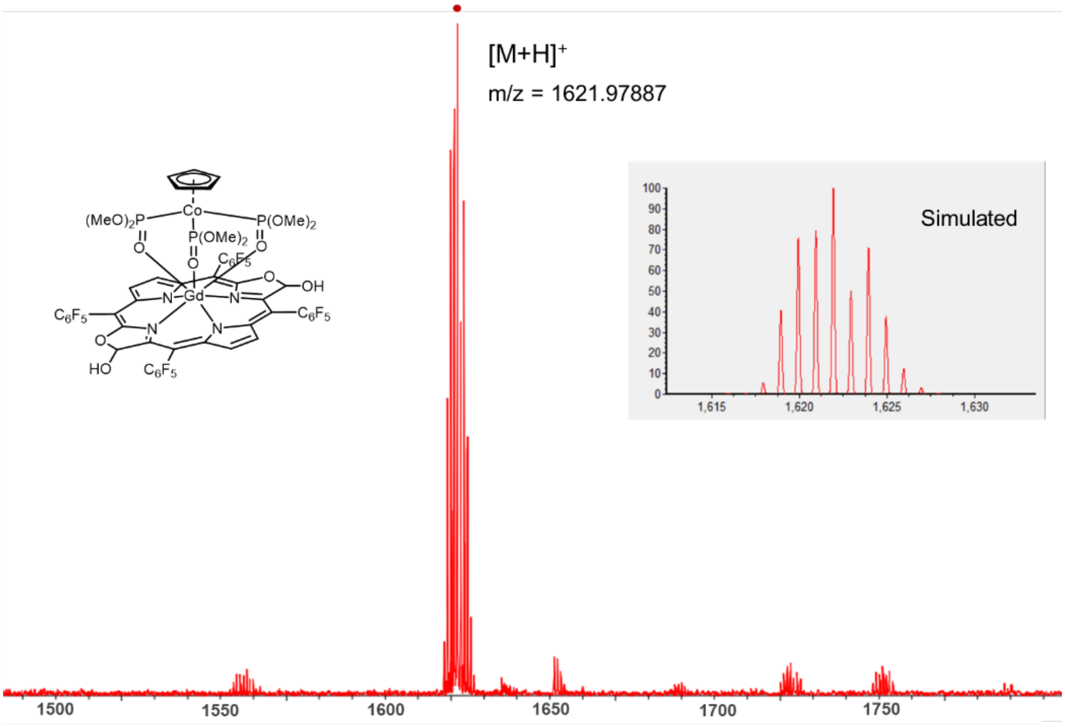

Fig. S31 HR-MS spectrum (ESI+) of Gd-*cis*-**2**. Inset shows the simulated spectrum.

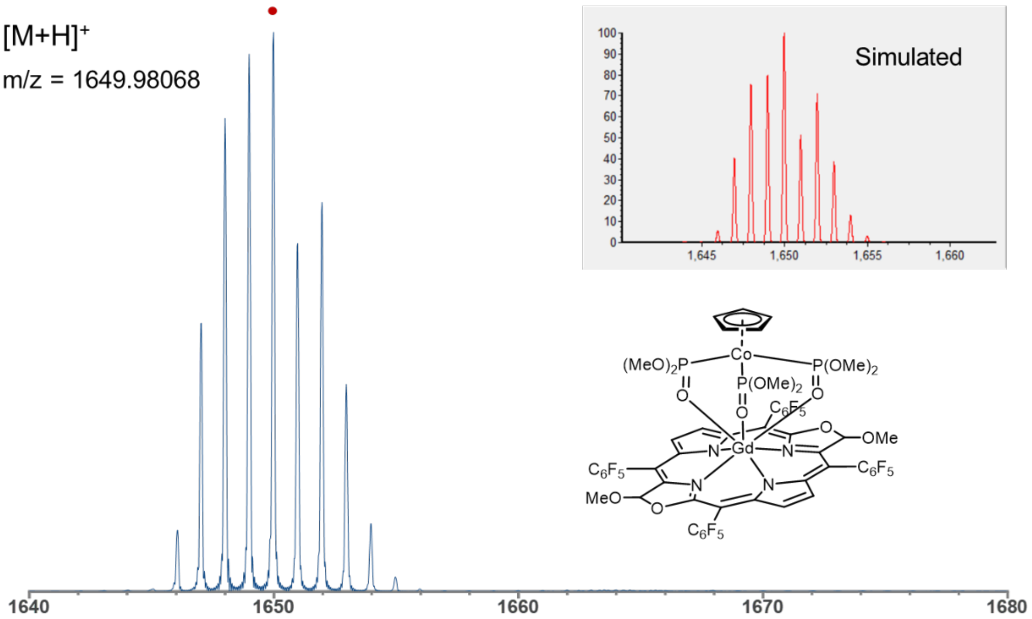

Fig. S32 HR-MS spectrum (ESI$^+$) of Gd-*trans*-**3**. Inset shows the simulated spectrum.

Table S2 F_{20}TPP S_0 optimised geometry.

C	2.45154400	-2.42879800	-0.00127300
C	-2.45108700	2.42833200	-0.00345400
N	2.03862400	-0.00016100	0.00588200
N	-2.03796000	-0.00038200	-0.00239500
C	2.86241200	-1.08441500	0.00205400
C	-2.86170700	1.08373500	-0.01776000
N	0.00007500	2.10272600	-0.00454200
N	0.00058000	-2.10328800	-0.00176600
C	4.26352500	-0.67498400	0.00734400
C	-4.26225000	0.67406000	-0.05704300
C	4.26345100	0.67475600	0.01351100
C	-4.26191200	-0.67566800	-0.06196600
C	2.86226900	1.08410700	0.01488200
C	-2.86118400	-1.08483300	-0.02571500
C	2.45105700	2.42845700	0.02599000
C	-2.45018500	-2.42921400	-0.02397500
C	1.12806700	2.88324100	0.02387900
C	-1.12736800	-2.88426100	-0.01236100

C	0.68265300	4.24493800	0.05934500
C	-0.68198900	-4.24643500	-0.01690400
C	-0.68307800	4.24494700	0.05180100
C	0.68380800	-4.24625300	-0.01348200
C	-1.12813700	2.88325800	0.01143700
C	1.12877100	-2.88398200	-0.00345300
C	3.51449400	-3.48318700	-0.00386800
C	-3.51389600	3.48250100	-0.00203400
C	3.91467100	-4.11322900	1.17404300
C	-3.87613200	4.15017500	-1.17169000
C	4.90283500	-5.09056000	1.18462200
C	-4.86390500	5.12783400	-1.18337300
C	5.51569800	-5.45690600	-0.00798600
C	-5.51541400	5.45656600	-0.00034900
C	5.13760400	-4.84740400	-1.19870400
C	-5.17586500	4.80932000	1.18201600
C	4.14589300	-3.87376900	-1.18432300
C	-4.18385400	3.83600200	1.16905200
C	3.51343700	3.48307500	0.04571800
C	-3.51318500	-3.48337200	-0.03970100
C	3.90626200	4.13934400	-1.12040900
C	-4.16450600	-3.87354000	1.13008100
C	4.89373200	5.11741700	-1.11537600
C	-5.15679200	-4.84672200	1.12790600
C	5.51345000	5.45801800	0.08129700
C	-5.51535800	-5.45603400	-0.06891800
C	5.14281300	4.82219500	1.26053500
C	-4.88252800	-5.09001200	-1.25115900
C	4.15174500	3.84829700	1.23067200
C	-3.89414400	-4.11326500	-1.22408800
F	3.34098500	-3.78161900	2.33677900
F	-3.26548100	3.85524600	-2.32552600
F	3.81608600	3.25266900	2.38092100
F	-3.30122800	-3.78181800	-2.37732400
F	5.26726900	-5.67753900	2.32788800

F	-5.19158400	5.75053900	-2.31884000
F	6.46498000	-6.39291700	-0.00995900
F	-6.46467700	6.39257100	0.00046800
F	5.72669200	-5.20194300	-2.34395600
F	-5.80159800	5.12803100	2.31831100
F	3.80299600	-3.30336900	-2.34511700
F	-3.87860000	3.22963800	2.32203700
F	3.32621500	3.83291400	-2.28684300
F	-3.84071700	-3.30351400	2.29638200
F	5.25113000	5.72940800	-2.24766200
F	-5.76494800	-5.20103700	2.26316700
F	6.46210200	6.39450300	0.09822400
F	-6.46502500	-6.39154700	-0.08279900
F	5.73823700	5.15216600	2.40987100
F	-5.22832000	-5.67665000	-2.40042100
H	5.12013900	-1.33209600	0.00898700
H	-5.11856200	1.33098100	-0.08433100
H	5.12004700	1.33187900	0.01425400
H	-5.11786100	-1.33292400	-0.09291100
H	-1.33121600	-5.10782600	-0.02209800
H	-1.33248100	5.10580100	0.07837600
H	1.33333500	-5.10741200	-0.02048400
H	1.33180000	5.10576100	0.09264100
H	0.00020700	1.09114000	-0.02593600
H	0.00043300	-1.09153100	0.00991200

Table S3 F_{20}TPP S_1 optimised geometry.

C	2.47208200	2.42558500	0.00437000
C	-2.46980300	-2.42398000	0.01079400
N	2.05885100	-0.00934600	-0.00214100
N	-2.05640500	0.01113000	-0.00093400
C	2.89277000	1.07477300	0.00956600
C	-2.88950300	-1.07264400	0.03905300
N	-0.00880500	-2.10391700	0.02592600
N	0.01162100	2.10477700	-0.00338000

C	4.28378600	0.65785700	0.00852700
C	-4.27788600	-0.65555600	0.12407100
C	4.27723600	-0.69781800	-0.00211900
C	-4.27090200	0.70020600	0.12840900
C	2.88225700	-1.10125900	-0.01477300
C	-2.87825400	1.10336000	0.04685600
C	2.44820200	-2.44789700	-0.03820100
C	-2.44489400	2.45025400	0.03639800
C	1.11767200	-2.89164600	-0.03568200
C	-1.11505500	2.89460400	0.01197000
C	0.66352700	-4.24834200	-0.11840500
C	-0.66115100	4.25403100	0.01192200
C	-0.70426100	-4.24171000	-0.10637500
C	0.70665100	4.24691000	0.00891100
C	-1.14380400	-2.88070500	-0.01579500
C	1.14642500	2.88288800	-0.00273400
C	3.53418800	3.47570200	0.00738500
C	-3.53161900	-3.47311600	0.00661200
C	3.84857400	4.19748300	-1.14500600
C	-3.79097400	-4.25345100	1.13489000
C	4.83915200	5.17216200	-1.15469400
C	-4.78154500	-5.22799900	1.14344700
C	5.54504200	5.44289200	0.01158400
C	-5.54383100	-5.44010400	0.00077400
C	5.25580900	4.74086800	1.17609000
C	-5.31048300	-4.67961800	-1.13933000
C	4.25966500	3.77230300	1.16233400
C	-4.31376500	-3.71178400	-1.12519400
C	3.49857300	-3.50842900	-0.07556000
C	-3.49774700	3.50908700	0.06303500
C	3.79211700	-4.28287300	1.04814400
C	-4.26077200	3.80155700	-1.06873000
C	4.77149000	-5.26857200	1.02656100
C	-5.25008700	4.77713800	-1.05609600
C	5.48691800	-5.49791200	-0.14276400

C	-5.49471300	5.48999000	0.11204200
C	5.21834100	-4.74359300	-1.27920300
C	-4.75132700	5.22313800	1.25570800
C	4.23344400	-3.76461900	-1.23457000
C	-3.76819200	4.24162400	1.21986600
F	3.18794100	3.95879900	-2.28444400
F	-3.07772600	-4.07103300	2.25279600
F	3.99570400	-3.05834800	-2.34597400
F	-3.07179200	4.00615400	2.33873900
F	5.11900400	5.84596400	-2.27363800
F	-5.00854600	-5.95685700	2.23962400
F	6.49747200	6.37545800	0.01392100
F	-6.49657700	-6.37227800	-0.00240800
F	5.93199300	5.00509400	2.29730600
F	-6.03992300	-4.88836500	-2.23861600
F	4.00286100	3.11510500	2.29929000
F	-4.11069000	-3.00007800	-2.23970400
F	3.12304400	-4.08464300	2.19024600
F	-4.04743500	3.13424200	-2.20841900
F	5.03192500	-5.99207100	2.11878200
F	-5.96217400	5.03778200	-2.15562100
F	6.42855800	-6.44077800	-0.17502600
F	-6.44032200	6.42919100	0.13492500
F	5.90302000	-4.96854700	-2.40386900
F	-4.98871700	5.90693600	2.37848900
H	5.14532000	1.30797900	0.00968300
H	-5.13723000	-1.30569700	0.18578800
H	5.13243800	-1.35623900	0.00299200
H	-5.12349700	1.35892400	0.19286400
H	-1.30386000	5.11999700	0.01422200
H	-1.35619600	-5.09842800	-0.16990500
H	1.35843200	5.10599900	0.02108300
H	1.30602800	-5.11128900	-0.19284300
H	-0.00313800	-1.09433100	0.07161700
H	0.00618800	1.09430900	-0.02391500

Table S4 $F_{20}TPP$ T_1 optimised geometry.

C	2.50373800	-2.40098100	0.00485400
C	-2.50330300	2.39867600	0.01092100
N	2.06872500	0.01799100	0.00523800
N	-2.06746300	-0.01982800	0.00236000
C	2.90900000	-1.06557700	-0.00283900
C	-2.90811700	1.06274900	-0.01330600
N	-0.01994500	2.11454400	-0.01408000
N	0.02117700	-2.11599200	0.00678800
C	4.30630200	-0.63417900	-0.00957300
C	-4.30436900	0.63062300	-0.06674400
C	4.29372000	0.71230900	-0.00707700
C	-4.29073600	-0.71586500	-0.07802500
C	2.88867300	1.11760100	0.00802900
C	-2.88623300	-1.12021100	-0.03312300
C	2.45791600	2.44461700	0.02692900
C	-2.45546900	-2.44670300	-0.03645500
C	1.09751900	2.90990800	0.04749500
C	-1.09620700	-2.91348000	-0.01720300
C	0.65882800	4.23924200	0.14761700
C	-0.65772200	-4.24671300	-0.03167600
C	-0.73854400	4.22634700	0.14490600
C	0.73959100	-4.23385800	-0.02115100
C	-1.15225900	2.88908800	0.04293600
C	1.15329700	-2.89273300	0.00354300
C	3.56564500	-3.45266600	0.00469600
C	-3.56429100	3.45031000	0.01536900
C	3.92258000	-4.12032700	1.17586600
C	-3.86175900	4.18302800	-1.13390400
C	4.90266200	-5.10531200	1.18800500
C	-4.84046500	5.16919200	-1.14167700
C	5.54889300	-5.44269800	0.00426300
C	-5.54649700	5.44252600	0.02436000
C	5.21291100	-4.79576900	-1.17924300

C	-5.27097700	4.73057200	1.18585300
C	4.22913600	-3.81418000	-1.16716900
C	-4.28767600	3.74853900	1.16994600
C	3.49820700	3.51698000	0.04384700
C	-3.49780300	-3.51779100	-0.06590700
C	3.80255000	4.24695400	-1.10522000
C	-4.15777900	-3.91781900	1.09523600
C	4.76138800	5.25253900	-1.10216000
C	-5.12395200	-4.91680100	1.08133000
C	5.43947400	5.54856800	0.07485600
C	-5.44524100	-5.54158800	-0.11805500
C	5.15614100	4.83983600	1.23646200
C	-4.80200000	-5.16529500	-1.29167400
C	4.19302200	3.83825400	1.20957000
C	-3.83946100	-4.16380000	-1.25342900
F	3.31666900	-3.81446200	2.32881800
F	-3.19949300	3.93897400	-2.27040800
F	3.94092000	3.17239000	2.34271800
F	-3.23570800	-3.82007700	-2.39701800
F	5.22884100	-5.72610800	2.32460400
F	-5.10932500	5.85156300	-2.25778700
F	6.49128500	-6.38534100	0.00454500
F	-6.48805900	6.38591700	0.02791400
F	5.83461500	-5.12132600	-2.31558500
F	-5.94916600	4.99522000	2.30556000
F	3.92638200	-3.20655400	-2.32041000
F	-4.04370500	3.07945300	2.30283200
F	3.16623800	3.98170500	-2.25167900
F	-3.86863800	-3.33190100	2.26280700
F	5.03753000	5.93215900	-2.21812400
F	-5.74300800	-5.27973500	2.20767000
F	6.36182800	6.51066500	0.08892700
F	-6.37075100	-6.50051300	-0.14333200
F	5.80770500	5.12609100	2.36666400
F	-5.11408800	-5.76509300	-2.44348600

H	5.16999900	-1.28191000	-0.01228300
H	-5.16777800	1.27788100	-0.09984500
H	5.14521400	1.37589200	-0.01721000
H	-5.14071800	-1.38027900	-0.11927200
H	-1.29391400	-5.11718400	-0.04839300
H	-1.39070400	5.08205400	0.21690600
H	1.39181900	-5.09238500	-0.03731800
H	1.29479300	5.10683000	0.22222900
H	-0.01060100	1.10631400	-0.07613000
H	0.01179700	-1.10614000	0.03442700

Table S5 F_{20}TPPLac S_0 optimised geometry.

O	5.29510100	0.51961000	-0.10074800
O	3.90655000	-1.27386200	-0.07323100
N	0.15209200	2.07505300	0.00273400
N	1.88696100	-0.25065500	0.00244900
F	3.67603800	3.27637800	-2.37951500
F	5.84598200	4.89407300	-2.40655900
F	7.14316400	5.46957300	-0.08269400
F	6.25910500	4.42741400	2.27142100
F	4.09320500	2.80396700	2.30336400
F	2.40897000	-4.42849400	-2.26376500
F	4.20926500	-6.44087900	-2.30793200
F	5.77882500	-6.91616800	-0.13428400
F	5.53331400	-5.37031100	2.09246100
F	3.72714300	-3.36065600	2.15168600
C	-0.87587800	2.98712400	-0.00141000
C	-0.27156500	4.28232500	-0.01779600
H	-0.81114300	5.21625000	-0.02893700
C	1.08780000	4.11724700	-0.02069200
H	1.83816000	4.89182500	-0.03020800
C	1.36295600	2.71534800	-0.00985300
C	2.62960600	2.09935500	-0.01969800
C	2.83467200	0.72459300	-0.02007000
C	4.18071300	0.09107400	-0.06647300

C	2.54102600	-1.40829800	-0.02722000
C	2.03090000	-2.71265500	-0.02320600
C	3.81884500	3.00570000	-0.03497900
C	4.29574800	3.55183400	-1.22364300
C	5.41013800	4.37995700	-1.25290600
C	6.07379700	4.67358700	-0.06786900
C	5.61990900	4.14226100	1.13383500
C	4.50371700	3.31768300	1.13684100
C	3.00883200	-3.83664500	-0.05034800
C	3.16361900	-4.64350600	-1.17772900
C	4.08627000	-5.68147600	-1.21587300
C	4.89001500	-5.92373400	-0.10841800
C	4.76359300	-5.13267800	1.02795600
C	3.83076000	-4.10475800	1.04517700
N	-0.36705100	-2.10998900	0.00273000
N	-2.12455700	0.21738300	0.01030500
F	-3.81386500	-3.37496400	2.42159300
F	-5.96375400	-5.01234500	2.47695900
F	-7.31043900	-5.57191200	0.17817700
F	-6.49668100	-4.48579600	-2.18132300
F	-4.34936000	-2.84460300	-2.24645200
F	-2.93599000	4.14549100	2.34830400
F	-4.62634300	6.25554300	2.34728500
F	-5.76064500	7.09157500	0.01817500
F	-5.19783500	5.80665100	-2.31459400
F	-3.51124900	3.69373200	-2.32293400
C	0.66320000	-3.01065300	0.00382400
C	0.06664000	-4.31633700	0.04222600
H	0.61739500	-5.24337200	0.06196000
C	-1.28887800	-4.16324800	0.05903300
H	-2.02984800	-4.94675700	0.08613800
C	-1.58097400	-2.75868700	0.03642300
C	-2.83113600	-2.14773100	0.05159900
C	-3.07360800	-0.75468500	0.04217900
C	-4.41156500	-0.17920700	0.07830100

C	-2.81158300	1.39928600	0.02318500
C	-2.24290000	2.68368300	0.01056500
C	-4.01759600	-3.05995900	0.08553600
C	-4.45927000	-3.63273800	1.27771500
C	-5.56236700	-4.47713900	1.32129000
C	-6.25140800	-4.76370400	0.14847100
C	-5.83457200	-4.20777800	-1.05570200
C	-4.72785000	-3.36742700	-1.07458200
C	-3.17500500	3.85588200	0.01274000
C	-3.48174000	4.53614100	1.19050800
C	-4.34803000	5.62283000	1.20484400
C	-4.92855900	6.05077400	0.01637800
C	-4.64031600	5.39309900	-1.17403600
C	-3.77050400	4.30916800	-1.16373900
H	0.01686800	1.07332400	0.01720900
H	-0.27331400	-1.10341000	-0.01807500
C	-4.24799400	1.16215800	0.06552500
H	-5.34116100	-0.72685200	0.11451400
H	-5.01868500	1.91762700	0.08987700

Table S6 F_{20}TPPLac S_1 optimised geometry.

O	-5.29602500	-0.56319900	-0.15802600
O	-3.92173400	1.24325800	-0.10789400
N	-0.13037500	-2.07783000	0.00737400
N	-1.89818400	0.23544000	0.02625800
F	-3.43396600	-3.59848000	-2.30492800
F	-5.58297100	-5.23745900	-2.33562300
F	-7.08962700	-5.55214100	-0.08916300
F	-6.43485600	-4.22153700	2.19340000
F	-4.28989900	-2.57435900	2.23049200
F	-2.32788800	4.48577400	-2.24903500
F	-4.13816500	6.48360000	-2.33875000
F	-5.84361300	6.87751600	-0.25300600
F	-5.71954800	5.26443100	1.93591000
F	-3.89906600	3.27379100	2.04805500

C	0.90455000	-2.98323000	0.00384600
C	0.30964700	-4.28568900	-0.01066000
H	0.85634100	-5.21511300	-0.02537700
C	-1.05119000	-4.13253700	-0.00672900
H	-1.79261200	-4.91547700	-0.00625600
C	-1.34258600	-2.73100200	-0.00095200
C	-2.61486400	-2.13170900	-0.01168700
C	-2.83868500	-0.74563500	-0.01128700
C	-4.18267900	-0.12861300	-0.09572100
C	-2.56697200	1.39396000	-0.03102400
C	-2.06702200	2.70634500	-0.03879100
C	-3.79279100	-3.04741300	-0.03084000
C	-4.15559400	-3.74085100	-1.18422700
C	-5.25895100	-4.58381800	-1.21608800
C	-6.02978300	-4.74402100	-0.07124600
C	-5.69299100	-4.06478100	1.09421900
C	-4.58614500	-3.22908300	1.10113800
C	-3.05149100	3.81761300	-0.09307500
C	-3.14457100	4.66212700	-1.20175000
C	-4.07348500	5.69267000	-1.26418700
C	-4.94793400	5.89252900	-0.20253200
C	-4.88466300	5.06568600	0.91352300
C	-3.94469700	4.04558700	0.95703300
N	0.34488500	2.12073700	0.00601600
N	2.13540500	-0.20125000	0.00362600
F	3.62609900	3.58313600	2.40934200
F	5.75296400	5.24514300	2.49198600
F	7.25480800	5.65194100	0.25795600
F	6.61678500	4.38596900	-2.06597100
F	4.49189900	2.72069900	-2.16103200
F	2.76235800	-4.30238200	2.30020900
F	4.46771700	-6.39651200	2.30391500
F	5.82981100	-7.05125700	0.03939800
F	5.47655300	-5.59816000	-2.23523900
F	3.77419500	-3.50143700	-2.25142200

C	-0.69348200	3.01923900	-0.00044700
C	-0.10904700	4.32411500	0.04391400
H	-0.66390700	5.24847300	0.06378500
C	1.25366800	4.17957100	0.06713000
H	1.98744200	4.96944400	0.09442600
C	1.55410200	2.78027100	0.04788800
C	2.81882600	2.17599400	0.07055500
C	3.07777000	0.78464100	0.05598000
C	4.42015700	0.21832300	0.12047800
C	2.83665300	-1.37781000	0.03083000
C	2.27605200	-2.67320800	0.01723700
C	3.99222400	3.09869000	0.12099900
C	4.34592600	3.76468300	1.29536400
C	5.43701500	4.62338000	1.35312700
C	6.20595000	4.83125900	0.21389500
C	5.87935900	4.18262900	-0.97158000
C	4.78249700	3.33023300	-1.00588700
C	3.21488700	-3.83465500	0.02402600
C	3.41807500	-4.60187200	1.17214700
C	4.29139000	-5.68260000	1.18919900
C	4.98906400	-6.01747800	0.03437900
C	4.80845200	-5.27316000	-1.12577700
C	3.92820200	-4.19797200	-1.11949100
H	-0.00247800	-1.07578100	0.02160600
H	0.25581100	1.11434700	-0.01531200
C	4.26964600	-1.12448400	0.10372200
H	5.34338700	0.77387200	0.18264500
H	5.04669000	-1.87197000	0.15057700

Table S7 F_{20}TPPLac T_1 optimised geometry.

O	5.32099800	0.42296800	-0.07164400
O	3.88658900	-1.33711800	-0.03329400
N	0.18160900	2.08867900	0.01720100
N	1.87791500	-0.29592700	0.00709700

F	4.04850600	2.72693100	-2.43211000
F	6.22252400	4.33968000	-2.49992000
F	7.21194800	5.38639400	-0.19009900
F	6.01359100	4.82602700	2.18967400
F	3.83480100	3.21875500	2.26139000
F	2.27220100	-4.63778700	-2.09847700
F	4.04214200	-6.67294800	-2.05608700
F	5.68491300	-7.01125300	0.08833700
F	5.53895900	-5.30489900	2.20403600
F	3.75527100	-3.27496500	2.18342700
C	-0.82559100	3.02273600	-0.02667500
C	-0.20057400	4.29441200	-0.10650800
H	-0.71628400	5.23947800	-0.16774100
C	1.17162600	4.09546500	-0.11099600
H	1.93536000	4.85446800	-0.17087900
C	1.40659400	2.70905400	-0.03611800
C	2.68207000	2.04545200	-0.04255600
C	2.87055900	0.69145200	-0.02710600
C	4.19322900	0.03047700	-0.04730900
C	2.52079900	-1.43517500	0.00572700
C	1.99128300	-2.76513500	0.03049500
C	3.88005200	2.93904600	-0.08471100
C	4.51294200	3.24251700	-1.28687500
C	5.63167600	4.06237600	-1.33475400
C	6.13897200	4.59620700	-0.15569800
C	5.52579900	4.31035200	1.05825000
C	4.40696300	3.48809600	1.08070700
C	2.95479600	-3.89327400	0.04574000
C	3.05722300	-4.78563300	-1.02383600
C	3.96651500	-5.83574200	-1.01805800
C	4.80903100	-6.00750300	0.07372700
C	4.73490600	-5.13309500	1.15246300
C	3.81547200	-4.09429700	1.12776000
N	-0.40477200	-2.11533000	-0.01245900
N	-2.14330300	0.28148200	0.00830100

F	-4.40873100	-2.63502700	2.40458800
F	-6.53325100	-4.30504300	2.45182400
F	-7.34381600	-5.54498700	0.16813700
F	-6.01830800	-5.10815600	-2.16886900
F	-3.88779900	-3.44170200	-2.22464200
F	-2.66860900	4.42907500	2.22970000
F	-4.31312000	6.57000900	2.17887900
F	-5.64771700	7.21241100	-0.10521100
F	-5.32797300	5.69891500	-2.34569900
F	-3.68456700	3.55346500	-2.30615500
C	0.60918900	-3.04512300	0.05405000
C	-0.00955300	-4.31575700	0.17717100
H	0.51228100	-5.25498900	0.26804100
C	-1.38429500	-4.12049900	0.18296400
H	-2.14235900	-4.88213500	0.27226900
C	-1.62324800	-2.73891600	0.06653800
C	-2.90585900	-2.07160400	0.05333700
C	-3.13326500	-0.71673600	0.02910400
C	-4.45539900	-0.10129900	0.02756700
C	-2.80708000	1.44382200	-0.00243700
C	-2.21080600	2.74431400	-0.01796200
C	-4.09000300	-2.98555400	0.08627100
C	-4.78665200	-3.23213700	1.26838100
C	-5.88085800	-4.08853500	1.30733600
C	-6.29550100	-4.72326100	0.14228000
C	-5.61695300	-4.49958000	-1.05070000
C	-4.52579300	-3.63968500	-1.06555000
C	-3.12221400	3.92507000	-0.03847100
C	-3.30928400	4.72216700	1.09209300
C	-4.15230000	5.82707400	1.08098600
C	-4.83582200	6.15593200	-0.08379900
C	-4.67233800	5.38105500	-1.22654700
C	-3.82192500	4.28318400	-1.19189400
H	0.03035400	1.09205100	0.07931500
H	-0.27917500	-1.11577100	-0.09144300

C	-4.26186900	1.22988000	0.01263400
H	-5.39795200	-0.62731700	0.03511800
H	-5.01683300	2.00141300	0.01719500

Table S8 *trans*-**1** S_0 optimised geometry.

O	-5.40594900	-0.45592800	-0.11589300
O	-3.98833600	1.31521500	-0.06739100
N	-0.29749000	-2.09229600	-0.00194200
N	-1.98844100	0.25681700	0.00233400
F	-3.82107300	-3.21290800	-2.42790200
F	-6.02519500	-4.78367800	-2.48815600
F	-7.35016100	-5.35758000	-0.17988700
F	-6.46044100	-4.36204600	2.19189400
F	-4.26017800	-2.78490100	2.25657400
F	-2.44919100	4.46653400	-2.22617700
F	-4.22318900	6.50310700	-2.24750100
F	-5.78271700	6.97705600	-0.06684000
F	-5.55527400	5.40579800	2.14360200
F	-3.77662000	3.37030600	2.17895600
C	0.72010200	-3.01118700	-0.02072300
C	0.10319700	-4.30221700	-0.07412300
H	0.63661500	-5.23880800	-0.11079200
C	-1.25327800	-4.12553000	-0.08193000
H	-2.00920300	-4.89395700	-0.11819100
C	-1.51931400	-2.72079900	-0.03916200
C	-2.76817700	-2.08535500	-0.04726800
C	-2.95028600	-0.70011100	-0.03211300
C	-4.28495100	-0.04555700	-0.07509600
C	-2.62261000	1.42954200	-0.01820800
C	-2.09169200	2.72473900	-0.00387500
C	-3.97502500	-2.96783100	-0.08139200
C	-4.45500700	-3.48909200	-1.27983400
C	-5.58675800	-4.29253100	-1.32605600
C	-6.26458200	-4.58533100	-0.14871300
C	-5.80771800	-4.07793400	1.06239800

C	-4.67416000	-3.27784400	1.08242700
C	-3.05517900	3.86173600	-0.01824600
C	-3.19932400	4.68179900	-1.13740300
C	-4.10866400	5.73175400	-1.16367800
C	-4.90753400	5.97326100	-0.05232000
C	-4.79046900	5.16908800	1.07605800
C	-3.87206700	4.12826900	1.08129100
O	5.40594800	0.45592800	0.11589800
O	3.98833600	-1.31521500	0.06740600
N	0.29749000	2.09229600	0.00196300
N	1.98844000	-0.25681700	-0.00228500
F	3.82106300	3.21293300	2.42792100
F	6.02518600	4.78370100	2.48816700
F	7.35016200	5.35757800	0.17989600
F	6.46045000	4.36202000	-2.19187800
F	4.26018600	2.78487600	-2.25654900
F	2.44919300	-4.46657700	2.22615200
F	4.22319200	-6.50314900	2.24743600
F	5.78271900	-6.97705500	0.06676500
F	5.55527400	-5.40575500	-2.14364600
F	3.77662000	-3.37026300	-2.17896000
C	-0.72010300	3.01118600	0.02073100
C	-0.10319800	4.30221800	0.07411600
H	-0.63661500	5.23880900	0.11077200
C	1.25327600	4.12553000	0.08192900
H	2.00920200	4.89395700	0.11818400
C	1.51931300	2.72079900	0.03917900
C	2.76817500	2.08535500	0.04729500
C	2.95028500	0.70011100	0.03215600
C	4.28494900	0.04555700	0.07516000
C	2.62260800	-1.42954200	0.01823600
C	2.09169200	-2.72473800	0.00388400
C	3.97502300	2.96783200	0.08141400
C	4.45500200	3.48910400	1.27985200
C	5.58675300	4.29254300	1.32607100

C	6.26458200	4.58532900	0.14872700
C	5.80772300	4.07792100	-1.06238100
C	4.67416400	3.27783200	-1.08240600
C	3.05518000	-3.86173500	0.01823200
C	3.19932500	-4.68182100	1.13737400
C	4.10866600	-5.73177500	1.16362800
C	4.90753500	-5.97326000	0.05226500
C	4.79047000	-5.16906600	-1.07609800
C	3.87206800	-4.12824600	-1.08131000
H	-0.17185700	-1.09115600	0.03696900
H	0.17185600	1.09115600	-0.03693800

Table S9 *trans*-**1** S_1 optimised geometry.

O	-5.39746800	-0.51300900	-0.14625800
O	-4.00226000	1.27418100	-0.08267000
N	-0.26555400	-2.10174300	0.00073100
N	-1.98808700	0.23936700	0.01887600
F	-3.65373400	-3.41866800	-2.40107700
F	-5.82794200	-5.02668600	-2.47203500
F	-7.26954400	-5.46832800	-0.20466800
F	-6.52637700	-4.29869200	2.13792300
F	-4.35689600	-2.68263200	2.21413700
F	-2.40175700	4.52760000	-2.18913800
F	-4.19205800	6.54608300	-2.23240200
F	-5.87044800	6.92920900	-0.12310300
F	-5.74030500	5.28509500	2.04214300
F	-3.94003800	3.27320600	2.10699300
C	0.75962600	-3.01631400	-0.02174100
C	0.15395700	-4.31070300	-0.08122300
H	0.69370900	-5.24320500	-0.12461200
C	-1.20675500	-4.14388500	-0.08496500
H	-1.95573800	-4.91895500	-0.12053900
C	-1.48452900	-2.74136400	-0.03830200
C	-2.74507600	-2.11806200	-0.04669300
C	-2.94112000	-0.73140000	-0.02642800

C	-4.27971500	-0.09345300	-0.08907500
C	-2.64546100	1.40817200	-0.01533800
C	-2.13629500	2.71706700	-0.00728100
C	-3.93860900	-3.01475600	-0.08748600
C	-4.34521000	-3.62586600	-1.27158300
C	-5.46095000	-4.45133900	-1.32370100
C	-6.19828100	-4.67670700	-0.16757100
C	-5.81621000	-4.08004500	1.02877700
C	-4.69729800	-3.26033100	1.05528400
C	-3.11071800	3.83840400	-0.03548500
C	-3.20627600	4.69877800	-1.13157900
C	-4.12444300	5.74004900	-1.16980900
C	-4.98504000	5.93461400	-0.09572000
C	-4.91862900	5.09207000	1.00844200
C	-3.98908900	4.06166300	1.02777800
O	5.39746900	0.51300700	0.14619700
O	4.00226200	-1.27418100	0.08259800
N	0.26555800	2.10174200	-0.00085900
N	1.98809900	-0.23937100	-0.01915100
F	3.65375900	3.41855800	2.40098300
F	5.82795900	5.02658500	2.47199300
F	7.26953600	5.46834100	0.20463200
F	6.52635200	4.29881200	-2.13800600
F	4.35687800	2.68274400	-2.21427500
F	2.40177900	-4.52733900	2.18929800
F	4.19207000	-6.54582400	2.23278800
F	5.87043700	-6.92921100	0.12351800
F	5.74027700	-5.28535900	-2.04192700
F	3.94001500	-3.27347500	-2.10700200
C	-0.75962200	3.01631200	0.02168200
C	-0.15395400	4.31069700	0.08124300
H	-0.69370600	5.24319600	0.12470100
C	1.20676000	4.14388000	0.08494900
H	1.95574300	4.91894900	0.12056000
C	1.48453400	2.74136200	0.03820000

C	2.74508200	2.11806000	0.04654500
C	2.94113000	0.73139800	0.02619600
C	4.27973000	0.09345100	0.08873800
C	2.64546800	-1.40817500	0.01518000
C	2.13629800	-2.71706900	0.00722600
C	3.93861400	3.01475500	0.08736900
C	4.34522300	3.62581300	1.27149100
C	5.46095900	4.45129000	1.32363600
C	6.19827700	4.67671600	0.16750900
C	5.81619800	4.08010700	-1.02886300
C	4.69728900	3.26038900	-1.05539800
C	3.11071800	-3.83840600	0.03555400
C	3.20628400	-4.69864800	1.13175000
C	4.12444800	-5.73991800	1.17009800
C	4.98503200	-5.93461600	0.09602400
C	4.91861300	-5.09220700	-1.00824000
C	3.98907600	-4.06179700	-1.02769100
H	-0.14394300	-1.10010600	0.04225300
H	0.14394800	1.10010700	-0.04244000

Table S10 *trans*-**1** T_1 optimised geometry.

O	-5.41759000	-0.45772100	-0.06150100
O	-3.98668100	1.30451100	-0.02671500
N	-0.28498400	-2.10880400	0.01076300
N	-1.97573900	0.26971600	0.00122700
F	-4.19447100	-2.69204700	-2.43656300
F	-6.36312800	-4.31161300	-2.50899000
F	-7.30101600	-5.43188300	-0.21270600
F	-6.05639600	-4.93927700	2.15808100
F	-3.88138800	-3.32660700	2.23399800
F	-2.42908600	4.60348600	-2.10831000
F	-4.22127600	6.61968400	-2.05922100
F	-5.84420300	6.95336400	0.10104100
F	-5.65635600	5.26189900	2.22534800
F	-3.85032400	3.25062800	2.19683300

C	0.73283800	-3.03329800	-0.04329800
C	0.11166200	-4.31541500	-0.14085500
H	0.63785400	-5.25367900	-0.21553400
C	-1.25596000	-4.12692700	-0.14487300
H	-2.01418700	-4.89043900	-0.21628800
C	-1.50452300	-2.73836400	-0.05347200
C	-2.78115200	-2.07891400	-0.05482100
C	-2.96652700	-0.72380900	-0.03201500
C	-4.29106900	-0.06362000	-0.04309700
C	-2.62284400	1.40554200	0.00516700
C	-2.10464400	2.74708500	0.02911200
C	-3.97717300	-2.97465800	-0.10069600
C	-4.63343600	-3.24466400	-1.29866100
C	-5.74981700	-4.06742200	-1.34866000
C	-6.23095200	-4.63895900	-0.17606500
C	-5.59423300	-4.38753300	1.03354100
C	-4.47818000	-3.56148800	1.05804500
C	-3.08057900	3.86546900	0.04746100
C	-3.20563400	4.74891600	-1.02675100
C	-4.12575300	5.78972500	-1.01735800
C	-4.95773600	5.95934600	0.08280400
C	-4.86183300	5.09282500	1.16635400
C	-3.93071200	4.06463700	1.13786200
O	5.41758900	0.45772500	0.06149400
O	3.98668100	-1.30450800	0.02670500
N	0.28498400	2.10880700	-0.01078100
N	1.97573800	-0.26971400	-0.00125800
F	4.19449100	2.69201000	2.43654100
F	6.36315000	4.31157400	2.50897500
F	7.30102000	5.43187900	0.21270200
F	6.05638000	4.93931000	-2.15808300
F	3.88137000	3.32664300	-2.23400700
F	2.42908100	-4.60345800	2.10832800
F	4.22127100	-6.61965700	2.05926700
F	5.84420100	-6.95336600	-0.10098900

F	5.65635800	-5.26192800	-2.22531800
F	3.85032700	-3.25065600	-2.19683100
C	-0.73283800	3.03329900	0.04329000
C	-0.11166200	4.31541500	0.14085900
H	-0.63785500	5.25367800	0.21554700
C	1.25596000	4.12692800	0.14487200
H	2.01418600	4.89043900	0.21629200
C	1.50452300	2.73836500	0.05345800
C	2.78115200	2.07891600	0.05480100
C	2.96652600	0.72381100	0.03198700
C	4.29106900	0.06362300	0.04305600
C	2.62284400	-1.40554000	-0.00518700
C	2.10464400	-2.74708400	-0.02911900
C	3.97717300	2.97465800	0.10068000
C	4.63344700	3.24464500	1.29864400
C	5.74982900	4.06740100	1.34864700
C	6.23095400	4.63895600	0.17605700
C	5.59422500	4.38755000	-1.03354700
C	4.47817200	3.56150600	-1.05805500
C	3.08057800	-3.86546800	-0.04745200
C	3.20563200	-4.74890200	1.02677200
C	4.12575000	-5.78971100	1.01739300
C	4.95773400	-5.95934700	-0.08276500
C	4.86183400	-5.09283900	-1.16632600
C	3.93071300	-4.06465100	-1.13784900
H	-0.15404900	-1.10948600	0.07712200
H	0.15404800	1.10948800	-0.07714700

Table S11 *trans*-**2** S_0 optimised geometry.

O	5.12972200	-0.37687100	-1.06425400
O	4.02438400	1.28567700	0.15644900
N	0.26257600	-2.09638500	-0.01032300
N	2.01550800	0.23990100	0.00795000
F	3.84057800	-3.09141700	2.49982800
F	5.99051600	-4.72890700	2.61105600

F	7.23021100	-5.50677900	0.31466900
F	6.31113600	-4.64029400	-2.09632700
F	4.15891800	-3.00902600	-2.21729700
F	2.47271900	4.50085700	2.22558900
F	4.26372800	6.51900300	2.22807000
F	5.87537800	6.91923200	0.06939200
F	5.67887800	5.28822300	-2.10207900
F	3.87987900	3.27383800	-2.12336100
C	-0.76777900	-3.00455500	0.00943900
C	-0.16598400	-4.29654700	0.09261900
H	-0.70791300	-5.22798500	0.13738500
C	1.19623300	-4.13305200	0.11870500
H	1.94135800	-4.91074600	0.17993200
C	1.47722700	-2.73661200	0.05612300
C	2.73615800	-2.10902800	0.07164300
C	2.95181700	-0.73508200	0.04685100
C	4.34534300	-0.11237600	0.05363500
C	2.66684200	1.40686100	0.06316100
C	2.13803100	2.70159000	0.03490100
C	3.93093200	-3.00877000	0.13666100
C	4.42910100	-3.46622300	1.35544900
C	5.53493600	-4.30488400	1.42881000
C	6.16893100	-4.70246100	0.25780300
C	5.69719400	-4.26007800	-0.97301200
C	4.59059700	-3.42342300	-1.01876800
C	3.11206100	3.82903300	0.04552600
C	3.24382900	4.67785900	1.14445100
C	4.16331900	5.71974100	1.16178200
C	4.98861800	5.92364900	0.06295100
C	4.88704300	5.08941800	-1.04466700
C	3.95683200	4.05963100	-1.04122200
O	-5.12972400	0.37686700	1.06425200
O	-4.02438400	-1.28567600	-0.15645400
N	-0.26257500	2.09638400	0.01032300
N	-2.01550800	-0.23990000	-0.00794900

F	-3.84058100	3.09142100	-2.49981900
F	-5.99052000	4.72891000	-2.61104100
F	-7.23021100	5.50677900	-0.31465000
F	-6.31113100	4.64029200	2.09634300
F	-4.15891200	3.00902500	2.21730600
F	-2.47270600	-4.50086300	-2.22559300
F	-4.26371500	-6.51900900	-2.22807900
F	-5.87537900	-6.91923200	-0.06941000
F	-5.67889300	-5.28821600	2.10205700
F	-3.87989400	-3.27383200	2.12334600
C	0.76777900	3.00455400	-0.00944100
C	0.16598400	4.29654600	-0.09262000
H	0.70791300	5.22798400	-0.13738700
C	-1.19623200	4.13305200	-0.11870100
H	-1.94135700	4.91074500	-0.17992600
C	-1.47722600	2.73661100	-0.05611900
C	-2.73615700	2.10902800	-0.07163700
C	-2.95181700	0.73508300	-0.04684800
C	-4.34534200	0.11237600	-0.05363600
C	-2.66684100	-1.40686100	-0.06316500
C	-2.13803100	-2.70159000	-0.03490600
C	-3.93093100	3.00877000	-0.13665200
C	-4.42910200	3.46622500	-1.35543800
C	-5.53493800	4.30488600	-1.42879600
C	-6.16893100	4.70246100	-0.25778700
C	-5.69719100	4.26007700	0.97302600
C	-4.59059400	3.42342100	1.01877800
C	-3.11206200	-3.82903300	-0.04553500
C	-3.24382200	-4.67786100	-1.14445800
C	-4.16331200	-5.71974400	-1.16179200
C	-4.98861900	-5.92364900	-0.06296600
C	-4.88705100	-5.08941500	1.04465000
C	-3.95684000	-4.05962800	1.04120800
H	0.14670600	-1.09546600	-0.06635500
H	-0.14670500	1.09546600	0.06635400

H	4.96433200	-0.37593100	0.91131100
H	4.60479100	-0.25432700	-1.86702900
H	-4.96433000	0.37593400	-0.91131200
H	-4.60479800	0.25430700	1.86702800

Table S12 *trans*-**2** S_1 optimised geometry.

O	-5.15554800	-0.47317800	1.01582400
O	-4.03812100	1.22726500	-0.13031200
N	-0.21859100	-2.10746300	0.01563000
N	-2.00815800	0.21530900	0.00900900
F	-3.74283200	-3.19175800	-2.50104900
F	-5.85538400	-4.87557800	-2.62240800
F	-7.12031400	-5.64123800	-0.33584700
F	-6.26456200	-4.71413900	2.07621900
F	-4.15020800	-3.03618700	2.20851100
F	-2.46579900	4.54305900	-2.17789800
F	-4.28087200	6.53624000	-2.19804700
F	-5.99207600	6.85103800	-0.10216600
F	-5.86354800	5.16064600	2.02937300
F	-4.03385000	3.17895700	2.07790000
C	0.82314300	-3.00728900	-0.02078800
C	0.23564600	-4.30385900	-0.12308000
H	0.78725600	-5.22848200	-0.18694900
C	-1.13062200	-4.15381000	-0.14133500
H	-1.86776500	-4.93825300	-0.21317900
C	-1.42618500	-2.76104200	-0.05890200
C	-2.70021200	-2.14973600	-0.06973700
C	-2.93140400	-0.77893600	-0.03322200
C	-4.33278600	-0.18141700	-0.06307300
C	-2.69121200	1.37435500	-0.04483500
C	-2.19478000	2.68814000	-0.02611700
C	-3.87789500	-3.06881000	-0.14136900
C	-4.34395200	-3.55900700	-1.36076900
C	-5.42993100	-4.42271000	-1.43955800
C	-6.07695400	-4.81421300	-0.27351700

C	-5.63755800	-4.34089400	0.95767400
C	-4.54940800	-3.48070200	1.00858900
C	-3.18495500	3.79503900	-0.04630800
C	-3.28191700	4.67863200	-1.12384400
C	-4.21389100	5.70841700	-1.15068700
C	-5.09094000	5.86826000	-0.08472000
C	-5.02528900	5.00232200	1.00089600
C	-4.07973500	3.98678400	1.00980100
O	5.15549000	0.47304400	-1.01589200
O	4.03812500	-1.22727900	0.13054200
N	0.21859200	2.10742500	-0.01538700
N	2.00816600	-0.21534000	-0.00897600
F	3.74323300	3.19180300	2.50072800
F	5.85573300	4.87574000	2.62167800
F	7.12022200	5.64140500	0.33488900
F	6.26410600	4.71419800	-2.07701400
F	4.14986600	3.03606100	-2.20888800
F	2.46517200	-4.54340300	2.17796900
F	4.28025900	-6.53655700	2.19838200
F	5.99210800	-6.85103600	0.10297900
F	5.86421300	-5.16034100	-2.02835700
F	4.03449500	-3.17868000	-2.07717000
C	-0.82313600	3.00726600	0.02091600
C	-0.23562400	4.30384800	0.12297700
H	-0.78722300	5.22847700	0.18683600
C	1.13064400	4.15379700	0.14109400
H	1.86779400	4.93824800	0.21277500
C	1.42620300	2.76101700	0.05878500
C	2.70022800	2.14970700	0.06960000
C	2.93142200	0.77890600	0.03322000
C	4.33279900	0.18137800	0.06309600
C	2.69121200	-1.37437600	0.04501300
C	2.19479200	-2.68817000	0.02637400
C	3.87790600	3.06880900	0.14103000
C	4.34414700	3.55905400	1.36033600

C	5.43009400	4.42281700	1.43891500
C	6.07689500	4.81432400	0.27275300
C	5.63731100	4.34095400	-0.95835400
C	4.54921500	3.48068200	-1.00904900
C	3.18496200	-3.79505900	0.04669900
C	3.28160400	-4.67881500	1.12413600
C	4.21358600	-5.70858900	1.15111600
C	5.09096500	-5.86826900	0.08539800
C	5.02563800	-5.00217200	-1.00011300
C	4.08007500	-3.98664600	-1.00916400
H	-0.10906800	-1.10578700	0.08200700
H	0.10906100	1.10576200	-0.08192100
H	-4.91043300	-0.43600500	-0.95304700
H	-4.65985800	-0.37689100	1.84060700
H	4.91052000	0.43606400	0.95299400
H	4.65981600	0.37651400	-1.84066000

Table S13 *trans*-**2** T_1 optimised geometry.

O	-5.20071300	-0.30822800	0.98030700
O	-3.98764300	1.31930600	-0.17371800
N	-0.27589000	-2.12634500	0.02910600
N	-1.98825700	0.27304700	-0.00681100
F	-3.79319500	-3.19317300	-2.47906900
F	-5.91016200	-4.87554900	-2.58857800
F	-7.22271700	-5.56190200	-0.30382900
F	-6.41460700	-4.55410900	2.09248500
F	-4.29980100	-2.87597200	2.21183900
F	-2.49081800	4.56217500	-2.26297900
F	-4.28963500	6.57265600	-2.23149300
F	-5.88826700	6.94416500	-0.05845000
F	-5.66742700	5.29442200	2.09735900
F	-3.85074800	3.29124300	2.08717000
C	0.74821000	-3.05101800	0.02956400
C	0.13607400	-4.32898900	-0.03688300
H	0.66240700	-5.27009200	-0.06315500

C	-1.24965800	-4.13950800	-0.07459800
H	-2.00259100	-4.90997500	-0.12479400
C	-1.48880000	-2.76764400	-0.03691100
C	-2.79741400	-2.07803600	-0.06924500
C	-2.98864600	-0.75459700	-0.04658500
C	-4.35701600	-0.08444100	-0.09035800
C	-2.64350700	1.38668900	-0.08419000
C	-2.12233800	2.75766600	-0.08175100
C	-3.98457400	-2.98544200	-0.13107000
C	-4.42289700	-3.51874100	-1.34220700
C	-5.50792600	-4.38368700	-1.41364100
C	-6.17902100	-4.73507000	-0.24808200
C	-5.76434600	-4.22052900	0.97495100
C	-4.67673600	-3.35892300	1.01872600
C	-3.10806900	3.86706800	-0.08336000
C	-3.25288900	4.72585100	-1.17362500
C	-4.17699500	5.76398300	-1.17336300
C	-4.99568900	5.95353000	-0.06703600
C	-4.88225800	5.10959300	1.03221700
C	-3.94516100	4.08686300	1.01207600
O	5.20288900	0.30925300	-0.97489400
O	3.98723400	-1.31957400	0.17452500
N	0.27573200	2.12603800	-0.03068600
N	1.98820500	-0.27324800	0.00391500
F	3.78601100	3.18992800	2.48513800
F	5.90268400	4.87214900	2.60277600
F	7.22165700	5.56141200	0.32259400
F	6.42030200	4.55670300	-2.07725400
F	4.30579200	2.87876000	-2.20471600
F	2.49088300	-4.56864500	2.25340400
F	4.29033900	-6.57836200	2.21651100
F	5.88966600	-6.94306000	0.04282200
F	5.66884400	-5.28711700	-2.10824700
F	3.85140700	-3.28464900	-2.09276400
C	-0.74835100	3.05072000	-0.03322900

C	-0.13630300	4.32875000	0.03285900
H	-0.66268800	5.26986300	0.05775200
C	1.24938800	4.13929200	0.07235500
H	2.00227500	4.90977500	0.12301400
C	1.48856300	2.76738900	0.03606500
C	2.79709300	2.07780000	0.07122300
C	2.98845400	0.75439900	0.04729700
C	4.35673300	0.08425400	0.09362100
C	2.64330900	-1.38693700	0.08171900
C	2.12224700	-2.75796200	0.07701200
C	3.98405100	2.98517300	0.13743900
C	4.41892000	3.51694600	1.35048100
C	5.50374800	4.38179400	1.42607400
C	6.17811600	4.73465000	0.26286500
C	5.76689900	4.22167100	-0.96199700
C	4.67940600	3.36012200	-1.00996600
C	3.10820800	-3.86711800	0.07582300
C	3.25315900	-4.72893600	1.16368300
C	4.17765300	-5.76672100	1.16066100
C	4.99672400	-5.95278600	0.05402700
C	4.88328300	-5.10570100	-1.04280600
C	3.94576200	-4.08343500	-1.01996500
H	-0.15034500	-1.12476600	0.05529100
H	0.15022000	1.12446200	-0.05724600
H	-4.93302400	-0.30566500	-0.98801700
H	-4.69808900	-0.30029100	1.80602600
H	4.93071700	0.30452800	0.99280700
H	4.70215900	0.30209400	-1.80177700

3. References

C. Lee, W. Yang and R. G. Parr, *Phys. Rev. B: Condens. Matter Mater. Phys.*, 1988, **37**, 785–789.

A. D. Becke, *J. Chem. Phys.*, 1993, **98**, 5648–5652.

M. J. Frisch, G. W. Trucks, H. B. Schlegel, G. E. Scuseria, M. A. Robb, J. R. Cheeseman, G. Scalmani, V. Barone, B. Mennucci, G. A. Petersson, H. Nakatsuji, M. Caricato, X. Li, H. P. Hratchian, A. F. Izmaylov, J. Bloino, G. Zheng, J. L. Sonnenberg, M. Hada, M. Ehara, K. Toyota, R. Fukuda, J. Hasegawa, M. Ishida, T. Nakajima, Y. Honda, O. Kitao, H. Nakai, T. Vreven, J. A. Montgomery, J. E. P. Jr., F. Ogliaro, M. Bearpark, J. J. Heyd, E. Brothers, K. N. Kudin, V. N. Staroverov, R. Kobayashi, J. Normand, K. Raghavachari, A. Rendell, J. C. Burant, S. S. Iyengar, J. Tomasi, M. Cossi, N. Rega, J. M. Millam, M. Klene, J. E. Knox, J. B. Cross, V. Bakken, C. Adamo, J. Jaramillo, R. Gomperts, R. E. Stratmann, O. Yazyev, A. J. Austin, R. Cammi, C. Pomelli, J. W. Ochterski, R. L. Martin, K. Morokuma, V. G. Zakrzewski, G. A. Voth, P. Salvador, J. J. Dannenberg, S. Dapprich, A. D. Daniels, Ö. Farkas, J. B. Foresman, J. V. Ortiz, J. Cioslowski and D. J. Fox, *Gaussian 09 (Revision E.01)*, Gaussian Inc., Wallingford CT, 2009.

M. M. Francl, W. J. Petro, W. J. Hehre, J. S. Binkley, M. S. Gordon, D. J. DeFree and J. A. Pople, *J. Chem. Phys.*, 1982, **77**, 3654-3665.

P. C. Hariharan and J. A. Pople, *Theor. Chem. Acc.*, 1978, **28**, 213.

P. v. R. Schleyer, C. Maerker, A. Dransfeld, H. Jiao and N. J. van Eikema Hommes, *J. Am. Chem. Soc.*, 1996, **118**, 6317-6318.

P. v. R. Schleyer, H. Jiao, N. J. v. E. Hommes, V. G. Malkin and O. L. Malkina, *J. Am. Chem. Soc.*, 1997, **119**, 12669-12670.

C. Brückner, J. R. McCarthy, H. W. Daniell, Z. D. Pendon, R. P. Ilagan, T. M. Francis, L. Ren, R. R. Birge and H. A. Frank, *Chemical Physics*, 2003, **294**, 285-303.

Y. H. Yao, Y. Rao, Y. W. Liu, L. Jiang, J. Xiong, Y. J. Fan, Z. Shen, J. L. Sessler and J. L. Zhang, *Phys. Chem. Chem. Phys.*, 2019, **21**, 10152-10162.

L. Huang, Y. Zhao, H. Zhang, K. Huang, J. Yang and G. Han, *Angew. Chem. Int. Ed.*, 2017, **56**, 14400-14404.

J. X. Zhang, H. G. Li, C. F. Chan, R. F. Lan, W. L. Chan, G. L. Law, W. K. Wong and K. L. Wong, *Chem. Commun.*, 2012, **48**, 9646-9648.

J. P. Tardivo, A. Del Giglio, C. S. de Oliveira, D. S. Gabrielli, H. C. Junqueira, D. B. Tada, D. Severino, R. D. F. Turchiello and M. S. Baptista, *Photodiagn Photodyn*, 2005, **2**, 175-191.

W. W. He, H. M. Jia, W. G. Wamer, Z. Zheng, P. J. Li, J. H. Callahan and J. J. Yin, *J Catal*, 2014, **320**, 97-105.

P. B. Momo, B. S. Bellete, T. J. Brocksom, R. O. M. A. de Souza and K. T. de Oliveira, *RSC Adv.*, 2015, **5**, 84350-84355.

Y. Yu, H. Lv, X. Ke, B. Yang and J. L. Zhang, *Adv. Synth. Catal.*, 2012, **354**, 3509-3516.

X. S. Ke, Y. Chang, J. Z. Chen, J. Tian, J. Mack, X. Cheng, Z. Shen and J. L. Zhang, *J. Am. Chem. Soc.*, 2014, **136**, 9598-9607.

Y. Y. Ning, Y. W. Liu, Y. S. Meng and J. L. Zhang, *Inorg. Chem.*, 2018, **57**, 1332-1341.
